# Genome-wide association and functional annotation analyses reveal candidate genes and pathways associated with various ewe longevity indicators in U.S. Katahdin sheep

**DOI:** 10.3389/fgene.2025.1600587

**Published:** 2025-07-03

**Authors:** Luis F. B. Pinto, Ronald M. Lewis, Artur O. Rocha, Brad A. Freking, Tom W. Murphy, Carrie S. Wilson, Sara M. Nilson, Joan M. Burke, Luiz F. Brito

**Affiliations:** ^1^ Department of Animal Science, Federal University of Bahia, Salvador, Brazil; ^2^ Department of Animal Sciences, Purdue University, West Lafayette, IN, United States; ^3^ Department of Animal Science, University of Nebraska-Lincoln, Lincoln, NE, United States; ^4^ United States Department of Agriculture - Agricultural Research Service, Roman L. Hruska U.S. Meat Animal Research Center, Clay Center, NE, United States; ^5^ United States Department of Agriculture - Agricultural Research Service, Range Sheep Production Efficiency Research Unit, Dubois, ID, United States; ^6^ United States Department of Agriculture - Agricultural Research Service, Dale Bumpers Small Farms Research Center, Booneville, AR, United States

**Keywords:** genes, genome-wide associate study, lifespan, longevity, ovine, productive life

## Abstract

Ewe longevity indicators are complex traits that are lowly heritable, expressed late in life, and sex-limited, making them challenging to include in breeding programs. In this context, genome-wide association studies (GWASs) can provide more information on the complex genetic control of these traits. Therefore, the primary objective of this study was to carry out association analyses for 8 longevity-related traits in 12,734 Katahdin ewes. A total of 126 associations at the chromosome-wide level and 3 at genome-wide level were found. These associations involved 86 single-nucleotide polymorphisms (SNPs) located across 22 chromosomes, with 24 of these SNPs associated with two or more traits. The variants overlapped with genes previously associated with prolificacy (*APOH*, *NLRP9*, *H3PXD2A*, *CKB*, and *HERC4*), ovarian follicle pool (*GALNT13*, *TMEM150B*, and *BRSK1*), synthesis and release of reproductive hormones (*SULT1B1*, *LEF1*, and *EIF5*), and early pregnancy events (*ITGAV*, *HADH*, *ZNFX1*, *ZSCAN4*, *EPN1*, *FBXW8*, *NOS1*, *ST3GAL4*, and *GFRA1*). Moreover, genes related to response to stress or pathological conditions (*ADCY5*, *HADH*, *ATRNL1*, *LEP*, *IL11*, *NLRP9*, *PRKCG*, *PRKCA*, *NEDD4L*, *FECH*, *CTNNA3*, *HECTD1*, *LRRTM3*, and zinc-finger proteins), growth performance (*GRID2*, *MED13L*, *DCPS*, and *LEP*), and carcass traits (*CMYA5* and *SETD3*) were also implicated. Metabolic pathways such as oxytocin signaling and cardiac-related pathways were enriched. These findings suggest that longevity indicators in Katahdin ewes are highly polygenic traits influenced by a combination of voluntary and involuntary culling reasons. Candidate genes and metabolic pathways influencing reproductive performance and health may play a key role in the functional longevity of Katahdin ewes.

## 1 Introduction

Ewe culling or death may be due to multiple causes, which can be classified as voluntary and involuntary culling (McLaren et al., 2020). Voluntary culling occurs when a breeder decides to cull a healthy and fertile ewe due to, for instance, low prolificity or old age. Voluntary culling is often necessary and contributes to genetic improvement of the flock as older ewes may be replaced with higher genetic merit ewe lambs. In contrast, involuntary culling occurs when a breeder culls (or the ewe dies) due to involuntary reasons such as disease or infertility, which results in ewe wastage. Involuntary culling is a major issue in many sheep flocks (Flay et al., 2021). Ewe wastage results in economic losses for the breeder due to the costs of raising more ewe lambs to maintain a consistent number of ewes in the flock, a reduction in the number of female lambs to sell, and loss of high production expected from mature ewes. A 10% reduction in ewe wastage could increase farm profitability by 17% (Farrell et al., 2019).

There are some direct indicators of ewe longevity, such as age at last lambing (ALL) and length of productive life (LPL), which measure total and functional lifetime, respectively. These traits have small additive genetic variances that can be used in selection schemes. For instance, heritability estimates of 0.05 for ALL were reported in both the U.S. Targhee sheep (Borg et al., 2009) and African Dorper sheep (Zishiri et al., 2013). In Spanish Churra dairy ewes, heritability estimates from 0.02 to 0.05 were reported for direct indicators of longevity (El-Saied et al., 2005). In New Zealand, longevity was defined as ALL minus 2 years, and heritability estimates of 0.10 (in ram breeder flocks) and 0.13 (in commercial flocks) were reported (Lee et al., 2015).

Accumulated ewe performance traits, such as the total number of litters (TNL), total number of lambs born (TNB), and weaned (TNW), as well as total litter body weight of the lamb measured at birth (TLB) and weaning (TLW), can be used as indirect longevity indicators. Furthermore, these traits seem to have slightly greater heritability estimates than ALL and LPL. For instance, heritability estimates of 0.10 (TNL), 0.11 (TNB), and 0.10 (TLW) were reported for Suffolk ewes (Milerski et al., 2018), while estimates of 0.13 (TLB) and 0.14 (TLW) were reported for Malpura sheep in India (Gowane et al., 2014). For South African Dorper sheep, low heritability estimates were reported for TNB (0.10) and TNW (0.09) (Zishiri et al., 2013), while moderate heritability estimates were reported for TNB (0.23), TNW (0.17), and TLW (0.20) in Merino sheep (Duguma et al., 2002). Therefore, genetic selection for improved longevity could significantly reduce ewe wastage.

Regardless of the differences in heritability estimates across studies, longevity-related traits in sheep have a heritable component that may be exploited in breeding schemes. However, many ewe longevity indicators tend to be recorded late in life and are sex-limited (female trait). Therefore, predicting accurate breeding values for selection candidates is difficult, especially for younger animals. Genomic selection has revolutionized genetic progress for such traits. However, in addition to performing genomic prediction of breeding values, there is a need to investigate the genomic architecture of complex traits such as ewe longevity indicators. Still, only one recent genome-wide association study (GWAS) investigated ewe longevity, which included Rambouillet, Polypay, Suffolk, and Columbia breeds (Smitchger et al., 2024). This study reported several single-nucleotide polymorphisms (SNPs) associated with traits directly and indirectly related to ewe longevity. Moreover, several genes previously associated with reproduction, dentition, and the immune system were reported. Nevertheless, this study was performed based on a small sample size (less than 500 animals per breed) from a single production system. Therefore, additional studies could validate the genomic regions previously reported or contribute to the identification of new genomic regions associated with ewe longevity.

The Katahdin is a composite sheep breed that is growing in popularity in the United States and has the largest reference population for genomic studies in the country. This breed has been directly selected for improving body weight at different ages, body composition, reproduction performance, and gastrointestinal parasite resistance (Notter and Lewis, 2018). However, U.S. Katahdin breeders have not directly selected for longevity indicators in breeding schemes. A previous study showed that a high percentage of primiparous ewes were culled before the second lambing (Pinto et al., 2025a). Then, variance components were estimated, and genomic predictions were carried out for eight longevity indicators in U.S. Katahdin sheep (Pinto et al., 2025b), which can be used as selection criteria to improve the U.S. Katahdin ewe longevity, but the genetic architecture of these traits remained unknown. Previous studies were performed to identify variants associated with resistance or susceptibility to gastrointestinal nematodes in Katahdin sheep (Becker et al., 2020; Becker et al., 2022; Notter et al., 2022). However, no previous studies were carried out to identify genomic variants associated with longevity-related traits in Katahdin sheep. Thus, the primary objective of this study was to carry out GWAS analyses to identify SNPs associated with eight ewe longevity indicators in this breed.

## 2 Materials and methods

### 2.1 Ethics approval

Approval from the Animal Use and Ethics Committee was not needed for this study as pre-existing datasets were provided by the National Sheep Improvement Program (NSIP) and recorded by U.S. Katahdin producers during routine husbandry activities.

### 2.2 Population and traits

The complete Katahdin pedigree from the NSIP contained information from 127,535 lambs born between 1985 and 2023. A total of 26,392 unique ewes with lambing records were identified, producing 118,510 lambs in 67,397 litters. Ewes (n = 1,233) with lambing intervals shorter than 150 days or longer than 720 days and ewes (n = 2,666) with age at first lambing younger than 270 days or older than 1,095 days were removed from further analyses. After this data filtering, 22,493 ewes from 231 flocks, which produced 100,981 lambs in 57,436 litters, remained.

We then removed flocks with few years of recorded data, inconsistent data reporting, or few records submitted to NSIP per year. Only data from flocks that reported lambing events for more than six consecutive years were retained. This threshold ensured that each flock was enrolled in NSIP long enough to capture the upper end of the expected productive life of ewes (McLaren et al., 2020; Hanna et al., 2023). Some flocks reported lambing records for more than 6 years but with a subsequent large and abrupt reduction in submitted lamb records in the last few years before leaving the NSIP; these were considered inconsistent flocks. We calculated the average number of lambing records across years for each flock. We found 20 lambing records per year to be the minimal value for excluding all the inconsistent flocks. This filter also enabled the exclusion of very small flocks, which reduced overall environmental noise. After the flock filters, data from 17,712 ewes from 58 flocks remained to be analyzed. These ewes produced 85,591 lambs in 48,533 litters.

Currently, the NSIP members do not record culling reasons and culling dates for all or the majority of their ewes. This prevents us from including the culling types (voluntary or involuntary) as a classification factor in our analyses, as done in studies for other species such as cattle (Oliveira et al., 2020). We recommend that sheep breeders start recording such information to facilitate future studies on ewe longevity based on culling reasons. As a consequence of the lack of this information in the studied population, we assumed that ewes were dead or culled if they had no lambing records during the last 2 years for which the flock reported data. Thus, ultimately, 12,734 ewes born between 1989 and 2020 in 58 flocks were identified as culled and included in subsequent GWAS analyses. The ewes contributing records were daughters of 1,245 sires and 6,325 dams and produced 61,178 lambs in 34,796 litters.

Eight longevity indicator traits were derived, and their definitions and abbreviations are presented in Table 1. Individual body weights at birth, weaning, and post-weaning required adjustment before calculating TLB, TLW, and an adjusted TLW (TLWadj). Lamb birth weight was adjusted to a female equivalent ( ${\text{BW}}_{\text{adj}}$ ). Lamb weaning weight (WW) was recorded between 30 and 90 days of age and adjusted to 60 days of age ( $\left.{\text{WW}}_{\text{adj}}\right)$ using the following equation:

$${\text{WW}}_{\text{adj}}=\left[\left(\frac{\text{WW}-\text{BW}}{N_{1}}\right)\times 60\right]+\text{BW},$$

where BW represents the lamb’s actual birth weight and $N_{1}$ represents the age at weaning in days. Lamb post-weaning weight (PWW) was recorded between 90 and 150 days of age and adjusted to 120 days of age ( ${\text{PWW}}_{\text{adj}}$ ) using the following equation:

$${\text{PWW}}_{\text{adj}}=\left[\left(\frac{\text{PWW}-\text{WW}}{N_{2}}\right)\times 60\right]+\text{WW},$$

where $N_{2}$ represents the number of days between weaning and post-weaning dates. ${\text{WW}}_{\text{adj}}$ and $P{\text{WW}}_{\text{adj}}$ were also adjusted to a female equivalent.

**TABLE 1:** Definition of indicator traits of ewe longevity.

Trait	Abbreviation	Definition
Age at last lambing	ALL	Ewe age (days) at last lambing
Length of productive life	LPL	Number of days between the first and last lambing
Total number of litters over ewe lifetime	TNL	Number of litters over the ewe lifetime
Total number of lambs born over ewe lifetime	TNB	Number of lambs born over the ewe lifetime
Total number of lambs weaned over ewe lifetime	TNW	Number of lambs weaned over the ewe lifetime
Total lamb birth weight over ewe lifetime	TLB	Sum of the body weight at lambing from all lambs produced over the ewe lifetime is measured in kg
Total lamb weight at weaning over ewe lifetime	TLW	Sum of the body weight at weaning from all lambs produced over the ewe lifetime is measured in kg
TLW divided by the ewe weight adjusted for 120 days	TLWadj	TLW divided by the ewe’s post-weaning body weight

The LPL can be defined as the number of days between the first lambing and culling (or death) (Ducrocq et al., 1988). As there were no records of culling or death dates in the Katahdin datasets provided by NSIP, we defined LPL as the difference (in days) between the first and last lambing records for each ewe (Milerski et al., 2018). For this reason, all ewes with LPL data had two or more lambing records. Descriptive statistics for all longevity indicator traits are presented in Table 2.

**TABLE 2:** Descriptive statistics for the longevity-related traits in U.S. Katahdin ewes.

Trait^a^	Sample size	Minimum	Maximum	Mean	Standard deviation
ALL (days)	12,538	270	3,239	1,102	636.5
LPL (days)	8,356	172	2,845	893.4	580.4
TNL	12,587	1	8	2.65	1.75
TNB	12,528	1	16	4.57	3.55
TNW	12,279	1	15	4.28	3.32
TLB (kg)	9,732	1.21	68.8	18.06	14.70
TLW (kg)	7,806	6.14	274.72	69.94	56.92
TLWadj (kg)	4,143	0.20	11.25	2.78	2.28

^a^ All abbreviations are presented in Table 1.

### 2.3 Genomic data

The NSIP Katahdin population had 10,032 animals genotyped. This population was genotyped with three SNP arrays: two versions of the Ovine 50K BeadChip and the Illumina Ovine Infinium HD BeadChip (Illumina Inc., San Diego, CA, United States). All SNP positions were remapped to the ARS-UI_Ramb_v2.0 reference using the National Center for Biotechnology Information Genome Remapping Service. Only shared positions were kept, and duplicate positions were reduced to those with the highest call rate. The distribution of genotyped animals across years is shown in Supplementary Figure S1. Quality control (QC) criteria were used to exclude SNPs with a minor allele frequency (MAF) lower than 0.05, SNPs and animals with a call rate lower than 0.90, SNPs with a difference greater than 0.15 between the observed and expected heterozygosity (extreme departure from Hardy–Weinberg equilibrium as an indication of genotyping errors), and non-autosomal SNPs. After QC, a panel of 30,408 SNPs genotyped in 10,032 animals remained for posterior analyses.

We also performed a principal component analysis of genomic information using the *plotPCA* option of postGSf90 software from the BLUPF90 suite (Misztal et al., 2022) to identify potential population stratification (Supplementary Figure S2). Supplementary Figure S2 is a Cartesian plot of the first two principal components of the genomic data of animals included in the study, which explains the largest percentage of total variance in the genotype data. A single cluster was observed and, therefore, we assumed no significant stratification in this population.

### 2.4 Association analyses

The GWAS analyses were performed based on the single-step GWAS (ssGWAS) approach (Misztal et al., 2009). The model can be described as follows:

y=Xβ+Zμ+e,

where y represents a vector $\left(n\times 1\right)$ of phenotypic values recorded in n ewes; β represents a $\left(p\times 1\right)$ vector of p fixed effects; μ represents a $\left(q\times 1\right)$ vector of q random effects; $X_{n\times p}$ and $Z_{n\times q}$ represent incidence matrices for fixed and random effects, respectively; and e represents a vector $\left(n\times 1\right)$ of residuals, which was assumed as $e\;∼\;N\left(0,I\sigma_{e}^{2}\right)$ , where I is an identity matrix and $\sigma_{e}^{2}$ is the residual variance. The mixed model equation (MME) can be described as follows:

$$\left[\begin{array}{cc} X^{\prime }X & \;X^{\prime }Z\\ Z^{\prime }X & \;Z^{\prime }Z+H^{-1}\lambda \end{array}\right]\left[\begin{array}{c} \hat{\beta }\\ \hat{\mu } \end{array}\right]=\left[\begin{array}{c} X^{\prime }y\\ Z^{\prime }y \end{array}\right].$$

In this MME, new terms include $\lambda =\frac{\sigma_{e}^{2}}{\sigma_{\mu }^{2}}$ , where $\sigma_{\mu }^{2}$ and $\sigma_{e}^{2}$ represent the additive genetic and residual variances, respectively, and $H^{-1}$ , which is the inverse of the hybrid relationship matrix as follows:

$$H^{-1}=A^{-1}+\left[\begin{array}{cc} 0 & \;0\\ 0 & \;G^{-1}-A_{22}^{-1} \end{array}\right],$$

where $A^{-1}$ represents the inverse of the numerator relationship matrix obtained from pedigree, $G^{-1}$ represents the inverse of the genomic relationship matrix (VanRaden, 2008), and $A_{22}^{-1}$ represents the inverse of the pedigree-based relationship matrix that includes only genotyped animals. The variance components were estimated using the Average Information Restricted Maximum Likelihood (AIREML) algorithm in the BLUPF90 suite (Misztal et al., 2022). First, renumf90 software was used to renumber and prepare the data and pedigree files, which were used as input in BLUPF90+ software (Misztal et al., 2022).

The animal effect was fitted as a random effect and assumed as $\mu \;∼\;N\left(0,{H\sigma }_{\mu }^{2}\right)$ . Ewe age at first lambing (≤378 days, 379–700 days, and >700 days) and birth-rearing type (1/1, 2/1, 2/2, 3/2, and 3/3) were fitted as categorical fixed effects. The contemporary group (CG; defined based on the flock-year-season of the ewe’s birth) was fitted as a random effect. The year-season effect was also included in the model as a fixed effect to avoid bias in the variance component estimation (Schaeffer, 2018). Only effects defining variation (P < 0.05) in a longevity-related trait based on ANOVA F-tests were included in the animal model fitted. Dam’s age class (≤713 days, 713 to 1,486 days, and >1,486 days) was tested as a categorical fixed effect in ANOVA, but it was not significant (P > 0.05) for any trait and, therefore, excluded. Phenotypic values outside three standard deviations from the mean were removed to avoid right skewness commonly observed in longevity traits. Based on graphical analyses, the residuals were assumed to fit a Gaussian distribution (Supplementary Figure S3). Our research group previously evaluated the model used in the current study, and further details can be found in Pinto et al. (2025b).

PostGSf90 software (Misztal et al., 2022) was used to estimate the SNP effect. The SNP effects were derived as $\hat{a}=\text{kD}Z^{\prime }G^{-1}\hat{\mu }$ , where $\hat{a}$ is a vector with estimates of SNP effects for each trait, $k=\frac{\sigma_{a}^{2}}{\sigma_{\mu }^{2}}$ corresponds to the ratio of SNPs to total additive direct variances, D is a diagonal matrix of SNP variances, **Z** is a centered matrix of allele content, and $\hat{\mu }$ is a vector of GEBV (Lourenco et al., 2020). The approximate p-value of i^th^ SNP was computed using ${\text{pval}}_{i}=2\left(1-\Phi \left|\frac{{\hat{a}}_{i}}{\text{sd}\left({\hat{a}}_{i}\right)}\right|\right)$ , where Φ is the cumulative standard normal function and $\text{sd}\left({\hat{a}}_{i}\right)$ is the square root of the prediction error variance (PEV) of the i^th^ SNP effect (Aguilar et al., 2019). Furthermore, the genomic inflation factor (Georgiopoulos and Evangelou, 2016) was calculated using R software version 4.3.1 (R Core Team, 2022). Then, chi-square statistics of each SNP tested were obtained using the {qchisq} function from R software version 4.3.1 (R Core Team, 2022). The chi-square statistic for each SNP tested was divided by the lambda value to calculate the chi-square adjusted for lambda. Finally, p-values of each chi-square adjusted for lambda were calculated using the {pchisq} function in R software version 4.3.1 (R Core Team, 2022). The p-values adjusted for lambda were posteriorly used to obtain the Manhattan plots in a –log10 (p-values adjusted for lambda) scale and the quantile–quantile plots. Both plot types were performed using the “QQman” R package version 0.1.9 (Turner, 2018).

The Bonferroni correction for multiple tests assumes that all hypothesis tests are independent. In the GWAS context, the association tests are not independent as there is linkage disequilibrium among SNPs. Therefore, significant threshold levels were calculated for each chromosome as $\propto_{i}=\frac{0.05}{Me_{i}}$ , where $\propto_{i}$ represents the threshold level for the i^th^ chromosome and ${Me}_{i}$ represents the number of independent segregant segments for the i^th^ chromosome. ${Me}_{i}$ was calculated using the “GALLO” R-package version 1.5 (Fonseca et al., 2020). For this, the length of each chromosome was defined based on the sheep RefSeq assembly GCF_016772045.2 (www.ncbi.nlm.nih.gov/), while the effective population size (Ne = 95) of the current Katahdin sheep population was calculated based on pedigree analysis using the “purgeR” package version 1.8.2 (López-Cortegano, 2022). The genome-wide threshold was calculated as $\propto =\frac{0.05}{\left(\sum_{i=1}^{n}Me_{i}\right)}$ , where n is the number of chromosomes in each analysis. The genome-wide threshold was 2.12 × 10^−05^, while the chromosome-wide threshold values per chromosome can be found in Supplementary Table S1.

### 2.5 Functional annotation analyses

The function {find_genes_qtls_around_markers} from the GALLO R-package version 1.5 (Fonseca et al., 2020) was used to identify candidate genes and quantitative trait loci (QTL) within a 100 Kbp upstream and 100 Kbp downstream region surrounding each significant SNP position. The gene map (ARS-UI_Ramb_v2.0.112. gtf) retrieved from the Ensembl database (https://ftp.ensembl.org/pub/release-112/gtf/ovis_aries_rambouillet/) and the QTL map (QTLdb_sheepOAR_rambo2. gff, release #53) retrieved from the Sheep QTLdb (www.animalgenome.org/cgi-bin/QTLdb/OA/index) were used as inputs in the GALLO R package version 1.5 (Fonseca et al., 2020). Functional genomic annotation and enrichment analyses were carried out on the DAVID (Database for Annotation, Visualization, and Integrated Discovery) web server (Sherman et al., 2022), while the STRING database (Szklarczyk et al., 2023) was used to analyze protein–protein association networks.

## 3 Results

### 3.1 Association analyses

**FIGURE 1:**
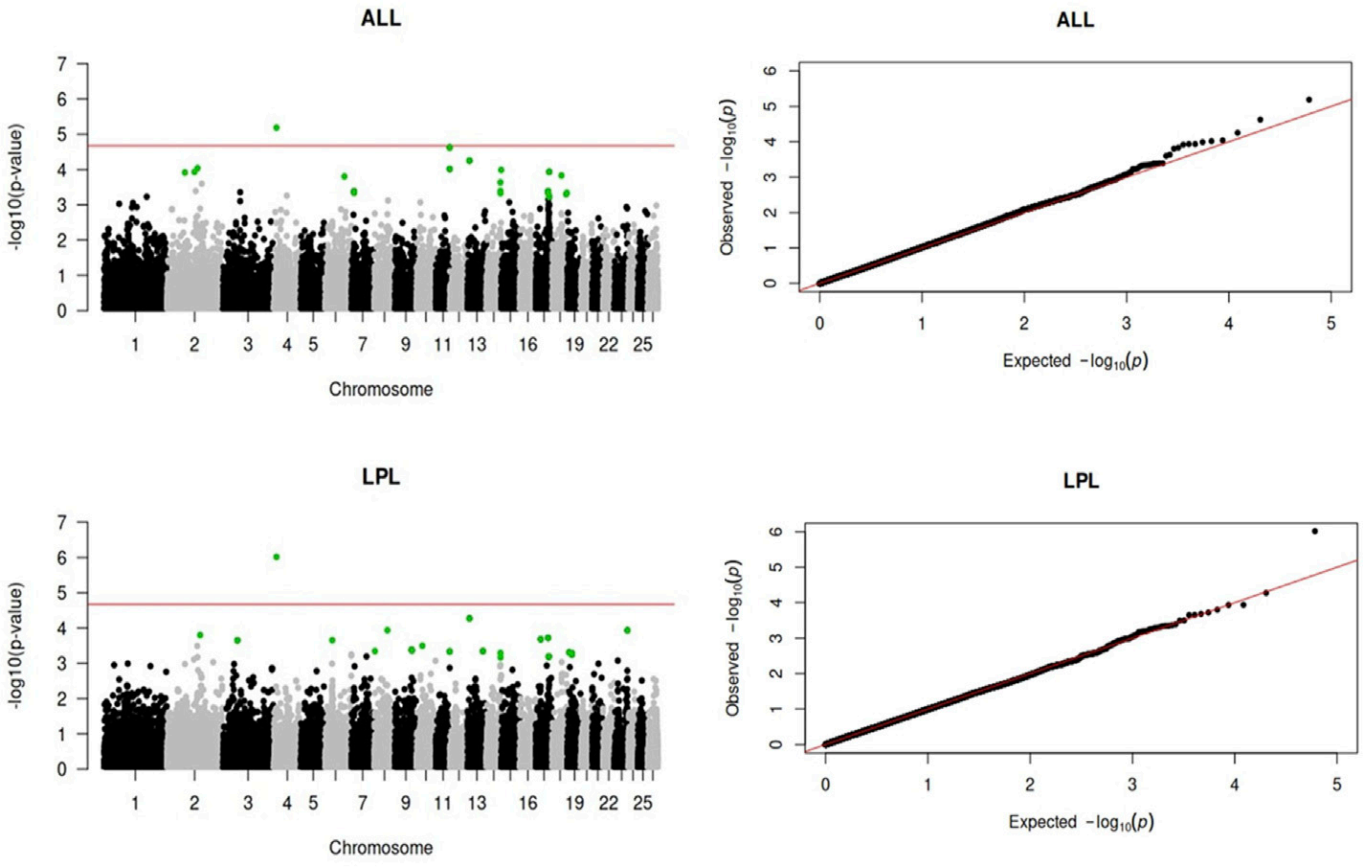
Manhattan and Q–Q plots of the genome-wide association analysis for age at last lambing (ALL) and length of productive life (LPL) in U.S. Katahdin sheep. The significant SNP at the chromosome-wide threshold are highlighted in green. The horizontal red line indicates the significance of the genome-wide threshold.

**FIGURE 2:**
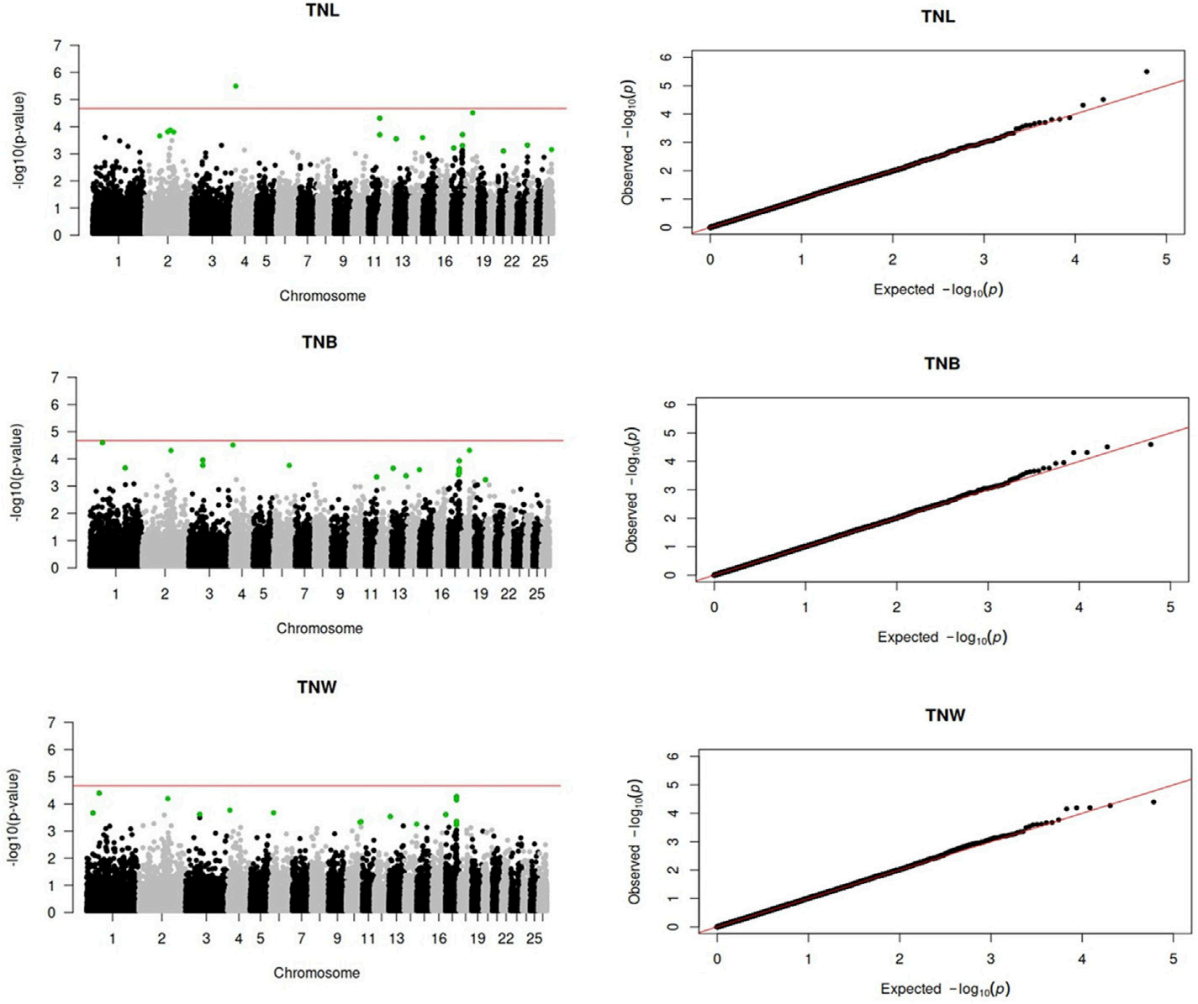
Manhattan and Q–Q plots of the genome-wide association analysis for total number of litters (TNL), lambs born (TNB), and lambs weaned (TNW) over ewe lifetime in U.S. Katahdin sheep. The significant SNP at the chromosome-wide threshold are highlighted in green. The horizontal red line indicates the significance of the genome-wide threshold.

**FIGURE 3:**
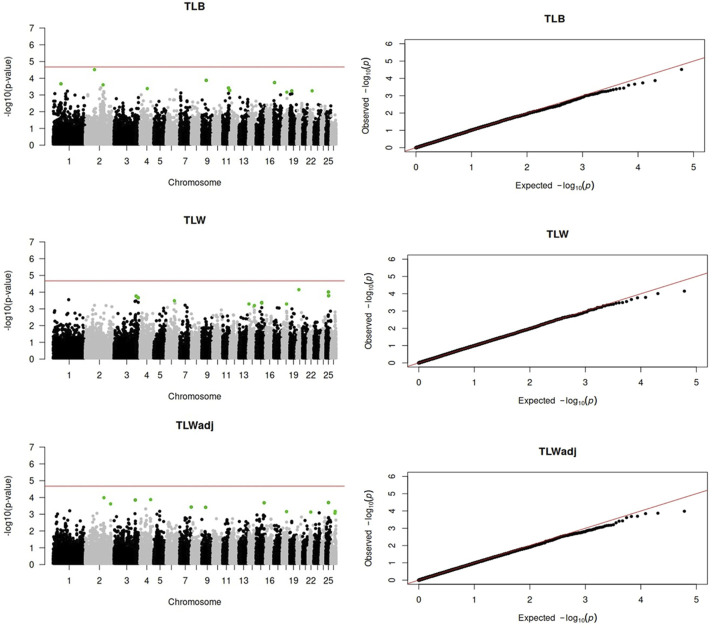
Manhattan and Q–Q plots of the genome-wide association analysis for total lamb weight at birth (TLB), total lamb weight at weaning (TLW), and total lamb weight at weaning divided by the ewe’s post-weaning weight (TLWadj) in U.S. Katahdin sheep. The significant SNP at the chromosome-wide threshold are highlighted in green. The horizontal red line indicates the significance of the genome-wide threshold.

Genome-wide association analyses were performed for eight longevity-related traits in Katahdin ewes. The distributions of the -log10 of p-values are presented in Figure 1 (ALL and LPL), Figure 2 (TNL, TNB, and TNW), and Figure 3 (TLB, TLW, and TLWadj). Green points in these figures indicate significant chromosome-wide associations, which were distributed as follows: 22 (ALL; Table 3), 22 (LPL; Table 4), 16 (TNL; Table 5), 18 (TNB; Table 6), 17 (TNW; Table 7), 11 (TLB; Table 8), 11 (TLW; Table 9), and 12 (TLWadj; Table 9). The number of significant associations per *Ovis aries* chromosomes (OAR) was as follows: 5 (OAR1), 14 (OAR2), 7 (OAR3), 7 (OAR4), 5 (OAR6), 3 (OAR7), 2 (OAR8), 4 (OAR9), 3 (OAR10), 8 (OAR11), 7 (OAR13), 11 (OAR14), 3 (OAR15), 25 (OAR17), 8 (OAR18), 5 (OAR19), 1 (OAR20), 1 (OAR21), 2 (OAR22), 2 (OAR23), 3 (OAR25), and 3 (OAR26). Moreover, the genome-wide threshold was indicated by a red horizontal line in Figures 1–3. Three genome-wide associations (P < 2.12 × 10^−05^) of the SNP rs399310772 with ALL, LPL, and TNL were found. This SNP is on 12.2 Mb of OAR4, has a MAF of 0.39, and is an intron variant in the novel genes (*ENSOARG00020028395* and *ENSOARG00020033537*). The Q–Q plots of each association analysis are also shown in Figures 1–3. The lambda values were approximately 1.00 for all traits as the p-values were adjusted for the genomic inflation factor.

**TABLE 3:** SNPs associated with the age at last lambing (ALL) in U.S. Katahdin sheep, including chromosome (OAR) position, genomic region, MAF, and p-value of the association test.

SNP	OAR	Position (bp)	Region^a^	MAF	SNP effect	P-value	Flanking gene (±100 kb)
rs400022476	2	80,179,672	Intergenic	0.09	0.2042	1.21 × 10^−04^	
rs402224636	2	122,777,052	Intron	0.32	−0.3864	1.16 × 10^−04^	*FAM171B* and ***ITGAV***
rs428593792	2	136,270,420	Intergenic	0.45	0.4175	9.15 × 10^−05^	
rs399310772	4	12,222,871	Intron	0.39	−0.4724	6.49 × 10^−06^ ^b^	*BET1*, ***ENSOARG00020028395***, and ***ENSOARG00020033537***
rs423900190	6	85,982,278	Missense	0.18	−0.3138	1.57 × 10^−04^	*LOC101119640*, ***SULT1B1***, *SULT1E1*, and *ENSOARG00020038822*
rs160278050	7	10,694,207	Missense	0.19	0.2883	4.51 × 10^−04^	*TENT2*, ***CMYA5***, *ENSOARG00020038995*, and *ENSOARG00020004170*
rs160278082	7	10,695,388	Missense	0.16	0.2624	4.13 × 10^−04^	*TENT2*, ***CMYA5***, *ENSOARG00020038995*, and *ENSOARG00020004170*
rs407234061	11	61,900,883	Intron	0.12	0.3075	2.37 × 10^−05^	***PRKCA*** and *APOH*
rs404999914	11	61,901,473	Intron	0.13	0.2874	9.63 × 10^−05^	***PRKCA*** and *APOH*
rs400995964	13	7,867,793	Intergenic	0.16	−0.3357	5.58 × 10^−05^	*ENSOARG00020025339*
rs411860439	14	62,375,192	Intergenic	0.10	−0.2463	2.31 × 10^−04^	*PRKCG*, *CACNG7*, *CACNG8*, *CACNG6*, *LOC101119153*, and *ENSOARG00020003572*
rs428707491	14	62,610,160	Intron	0.31	0.3524	4.06 × 10^−04^	*NLRP9*, *ENSOARG00020003899*, *U2AF2*, *CCDC106*, *LOC105612738*, *ZNF784*, *ZNF581*, *EPN1*, *ZNF580*, *ENSOARG00020038693*, *ENSOARG00020028170*, *ENSOARG00020037910*, ***ENSOARG00000000987***, and ***ENSOARG00000000997***
rs420711470	14	62,954,922	Intron	0.32	0.3422	4.69 × 10^−04^	*SHISA7*, *UBE2S*, *RPL28*, *TMEM190*, *IL11*, *COX6B2*, *KMT5C*, *TMEM150B*, *BRSK1*, *HSPBP1*, *PPP6R1*, *TMEM86B*, ***ENSOARG00000001564***, ***ENSOARG00000026944***, *ENSOARG00020005036*, *SYT5*, *DNAAF3*, *TNNI3*, *TNNT1*, *ENSOARG00020032367*, *ENSOARG00020038167*, *TMEM238*, *ENSOARG00020039845*, and *ENSOARG00020004991*
rs399288440	14	65,830,741	Intergenic	0.20	0.2874	1.03 × 10^−04^	*ZSCAN4*, *ENSOARG00020027141*, *LOC101110699*, *LOC101117358*, *LOC105601916*, *LOC101117613*, *LOC101110440*, and *ENSOARG00020008968*
rs416175412	17	54,837,118	Intergenic	0.18	−0.2954	4.13 × 10^−04^	*CCDC63*, *MYL2*, and *CUX2*
rs419277881	17	55,794,456	Intergenic	0.42	0.3756	4.63 × 10^−04^	*CIT*, *PRKAB1*, *TMEM233*, *CCDC60*, *ENSOARG00020034280*, and *ENSOARG00020031854*
rs418797399	17	58,814,585	Intron	0.48	−0.3529	5.82 × 10^−04^	***MED13L***
rs430801927	17	59,768,368	Intron	0.46	0.3603	5.93 × 10^−04^	***ENSOARG00020027578***, *ENSOARG00020028857*, *ENSOARG00020030413*, *ENSOARG00020030475*, *ENSOARG00020033868*, *ENSOARG00020037170*, and *ENSOARG00020040097*
rs405136354	17	59,793,006	Intergenic	0.43	0.4048	1.16 × 10^−04^	*ENSOARG00020027877*, *ENSOARG00020027578*, *ENSOARG00020028857*, *ENSOARG00020030413*, *ENSOARG00020030475*, *ENSOARG00020033868*, *ENSOARG00020037170*, and *ENSOARG00020040097*
rs416601769	18	41,773,186	Intron	0.39	0.3818	1.48 × 10^−04^	***NPAS3***
s37838.1	18	62,114,682	Intron	0.28	−0.3537	5.02 × 10^−04^	***SETD3***, *CCNK*, and *CCDC85C*
rs421227438	18	65,806,713	Intron	0.10	0.2184	4.59 × 10^−04^	*EIF5*, ***MARK3***, *CKB*, *TRMT61A*, *ENSOARG00020028257*, *ENSOARG00020028499*, *ENSOARG00020035051*, *ENSOARG00020035856*, and *ENSOARG00020014907*

^a^ The SNP located within genes had the genes highlighted in bold.

^b^ Significant SNPs at the genome-wide level.

**TABLE 4:** SNPs associated with the lenght of productive life (LPL) in U.S. Katahdin sheep, including chromosome (OAR) position, genomic region, MAF, and p-value of the association test.

SNP	OAR	Position (bp)	Region^a^	MAF	SNP effect	P-value	Flanking gene (±100 kb)
rs413377527	2	148,006,184	Intron	0.46	−0.4103	1.58 × 10^−04^	*SLC4A10* and ***ENSOARG00020012593***
rs425512192	3	63,753,099	Intergenic	0.26	0.3651	2.23 × 10^−04^	
rs399310772	4	12,222,871	Intron	0.39	−0.5204	9.62 × 10^−07^ ^b^	*BET1*, ***ENSOARG00020028395***, and ***ENSOARG00020033537***
rs400216417	6	32,071,830	Intron	0.46	0.3792	2.22 × 10^−04^	***GRID2***
rs409333251	8	2,269,243	Intron	0.09	−0.2115	4.52 × 10^−04^	***FILIP1***
rs402462960	8	57,802,965	Intron	0.21	−0.3552	1.16 × 10^−04^	***MOXD1***, *STX7*, *ENSOARG00020018939*, and *ENSOARG00020019065*
rs160663919	9	74,367,707	Intron	0.44	−0.3764	4.09 × 10^−04^	***ATP6V1C1***, *ENSOARG00020035917*, *ENSOARG00020031547*, and *ENSOARG00020020667*
rs418772518	9	75,310,128	Intergenic	0.42	−0.3810	4.32 × 10^−04^	*NCALD*
rs405467063	10	26,922,988	Intergenic	0.29	0.3438	3.19 × 10^−04^	
rs407234061	11	61,900,883	Intron	0.12	0.2562	4.64 × 10^−04^	***PRKCA*** and *APOH*
rs400995964	13	7,867,793	Intergenic	0.16	−0.3430	5.32 × 10^−05^	*ENSOARG00020025339*
rs408729143	13	67,728,289	Intron	0.06	0.1754	4.51 × 10^−04^	***PPP1R16B***, ***FAM83D***, *DHX35*, and *ENSOARG00020012310*
rs428707491	14	62,610,160	Intron	0.31	0.3537	5.09 × 10^−04^	*NLRP9*, *ENSOARG00020003899*, *U2AF2*, *CCDC106*, *LOC105612738*, *ZNF784*, *ZNF581*, *EPN1*, *ZNF580*, *ENSOARG00020038693*, *ENSOARG00020028170 ENSOARG00020037910*, ***ENSOARG00000000987***, and ***ENSOARG00000000997***
rs420711470	14	62,954,922	Intron	0.32	0.3430	6.80 × 10^−04^	*SHISA7*, *UBE2S*, *RPL28*, *TMEM190*, *IL11*, *COX6B2*, *KMT5C*, *TMEM150B*, *BRSK1*, *HSPBP1*, *PPP6R1*, *TMEM86B*, ***ENSOARG00000001564***, ***ENSOARG00000026944***, *ENSOARG00020005036*, *SYT5*, *DNAAF3*, *TNNI3*, *TNNT1*, *ENSOARG00020032367*, *ENSOARG00020038167*, *TMEM238*, *ENSOARG00020039845*, and *ENSOARG00020004991*
rs430305185	17	21,854,955	Intergenic	0.13	−0.2578	2.07 × 10^−04^	
rs416175412	17	54,837,118	Intergenic	0.18	−0.3157	1.90 × 10^−04^	*CCDC63*, *MYL2*, and *CUX2*
rs426404725	17	57,317,973	Intron	0.39	0.3412	6.52 × 10^−04^	***KSR2***
rs427138541	17	57,981,108	Intron	0.32	−0.3520	6.42 × 10^−04^	*NOS1*, *FBXO21*, ***TESC***, and *FBXW8*
rs418513057	19	8,561,369	Intergenic	0.24	−0.3155	4.92 × 10^−04^	*ENSOARG00020026694*
rs411226639	19	20,445,303	Intergenic	0.20	−0.3066	5.19 × 10^−04^	
rs423686700	19	20,695,001	Intergenic	0.14	−0.2644	5.64 × 10^−04^	
rs411106195	23	57,717,942	Intron	0.26	−0.3862	1.17 × 10^−04^	***NEDD4L***

^a^ The SNP located within genes had the genes highlighted in bold.

^b^ Significant SNPs at the genome-wide level.

**TABLE 5:** SNPs associated with the total number of litters (TNL) over ewe lifetime in U.S. Katahdin sheep, including chromosome (OAR) position, genomic region, MAF, and p-value of the association test.

SNP	OAR	Position (bp)	Region^a^	MAF	SNP effect	P-value	Flanking gene (±100 kb)
rs400022476	2	80,179,672	Intergenic	0.09	0.0006	2.19 × 10^−04^	
rs402224636	2	122,777,052	Intron	0.32	−0.0011	1.53 × 10^−04^	*FAM171B* and ***ITGAV***
rs428593792	2	136,270,420	Intergenic	0.45	0.0012	1.34 × 10^−04^	
rs426160726	2	155,017,143	Intergenic	0.26	0.0010	1.57 × 10^−04^	*GALNT13*, *ENSOARG00020036565*, and *ENSOARG00020026958*
rs399310772	4	12,222,871	Intron	0.39	−0.0014	3.20 × 10^−06^ ^b^	*BET1*, ***ENSOARG00020028395***, and ***ENSOARG00020033537***
rs407234061	11	61,900,883	Intron	0.12	0.0009	4.86 × 10^−05^	***PRKCA*** and *APOH*
rs404999914	11	61,901,473	Intron	0.13	0.0008	1.99 × 10^−04^	***PRKCA*** and *APOH*
rs400995964	13	7,867,793	Intergenic	0.16	−0.0009	2.79 × 10^−04^	*ENSOARG00020025339*
rs399288440	14	65,830,741	Intergenic	0.20	0.0008	2.53 × 10^−04^	*ZSCAN4*, *ENSOARG00020027141*, *LOC101110699*, *LOC101117358*, *LOC105601916*, *LOC101117613*, *LOC101110440*, and *ENSOARG00020008968*
rs429728006	17	13,031,302	Intergenic	0.43	−0.0011	6.08 × 10^−04^	*ENSOARG00020032263*
rs430801927	17	59,768,368	Intron	0.46	0.0011	5.01 × 10^−04^	***ENSOARG00020027578***, *ENSOARG00020028857*, *ENSOARG00020030413*, *ENSOARG00020030475*, *ENSOARG00020033868*, *ENSOARG00020037170*, and *ENSOARG00020040097*
rs405136354	17	59,793,006	Intergenic	0.43	0.0011	1.97 × 10^−04^	*ENSOARG00020027877*, *ENSOARG00020027578*, *ENSOARG00020028857*, *ENSOARG00020030413*, *ENSOARG00020030475*, *ENSOARG00020033868*, *ENSOARG00020037170*, and *ENSOARG00020040097*
rs416601769	18	41,773,186	Intron	0.39	0.0012	3.07 × 10^−05^	***NPAS3***
rs408858996	21	27,230,215	Intron	0.06	−0.0005	7.90 × 10^−04^	*DCPS*, *ST3GAL4*, and ***KIRREL3***
rs400623485	23	57,231,444	Intron	0.33	−0.0010	4.77 × 10^−04^	*FECH*, *NARS1*, ***ATP8B1***, *ENSOARG00020032445*, *ENSOARG00020033906,* and *ENSOARG00020024019*
rs430387043	26	37,823,010	Intron	0.29	−0.0009	6.98 × 10^−04^	***PSD3*** and *ENSOARG00020029860*

^a^ The SNP located within genes had the genes highlighted in bold.

^b^ Significant SNPs at the genome-wide level.

**TABLE 6:** SNPs associated with the total number of lambs born (TNB) over ewe lifetime in U.S. Katahdin sheep, including chromosome (OAR) position, genomic region, MAF, and p-value of the association test.

SNP	OAR	Position (bp)	Region^a^	MAF	SNP effect	P-value	Flanking gene (±100 kb)
rs425311695	1	66,241,221	Intron	0.48	0.0029	2.53 × 10^−05^	*KYAT3*, *LOC101119517*, *LOC101119773*, *LOC101120395*, *ENSOARG00020028768*, *ENSOARG00020029840*, and ***ENSOARG00020006716***
rs161707914	1	188,636,771	Intron	0.31	0.0023	2.16 × 10^−04^	*HACD2*, ***ADCY5***, and *ENSOARG00020032532*
rs426160726	2	155,017,143	Intergenic	0.26	0.0024	4.93 × 10^−05^	*GALNT13*, *ENSOARG00020036565*, and *ENSOARG00020026958*
rs408844024	3	76,243,463	Intergenic	0.44	−0.0026	1.73 × 10^−04^	*FOXN2*
rs419670964	3	76,250,495	Intergenic	0.43	−0.0027	1.11 × 10^−04^	
rs399310772	4	12,222,871	Intron	0.39	−0.0029	3.09 × 10^−05^	*BET1*, ***ENSOARG00020028395***, and ***ENSOARG00020033537***
rs423900190	6	85,982,278	missense	0.18	−0.0021	1.74 × 10^−04^	*LOC101119640*, ***SULT1B1***, *SULT1E1*, and *ENSOARG00020038822*
rs407234061	11	61,900,883	Intron	0.12	0.0016	4.63 × 10^−04^	***PRKCA*** and *APOH*
rs400995964	13	7,867,793	Intergenic	0.16	−0.0020	2.20 × 10^−04^	*ENSOARG00020025339*
rs416157010	13	77,378,009	Downstream	0.38	−0.0025	4.23 × 10^−04^	*KCNB1*, *STAU1*, *DDX27*, *ZNFX1*, ***ENSOARG00020034858***, ***ENSOARG00020009953***, ***ENSOARG00020009987***, and ***ENSOARG00020009998***
rs399288440	14	65,830,741	Intergenic	0.20	0.0017	2.51 × 10^−04^	*ZSCAN4*, *ENSOARG00020027141*, *LOC101110699*, *LOC101117358*, *LOC105601916*, *LOC101117613*, *LOC101110440*, and *ENSOARG00020008968*
rs424778250	17	55,644,362	Intron	0.34	−0.0023	3.81 × 10^−04^	***CIT***, *ENSOARG00020036135*, and ***ENSOARG00020019000***
rs405696543	17	59,616,349	Intergenic	0.45	0.0025	1.18 × 10^−04^	*ENSOARG00020028857*, *ENSOARG00020030475,* and *ENSOARG00020037170*
rs415377431	17	59,646,371	Intergenic	0.46	0.0024	3.22 × 10^−04^	*ENSOARG00020027578*, *ENSOARG00020028857*, *ENSOARG00020030475*, and *ENSOARG00020037170*
rs421430100	17	59,708,716	Intron	0.41	0.0024	2.84 × 10^−04^	*ENSOARG00020027578*, *ENSOARG00020028857*, ***ENSOARG00020030475***, and ***ENSOARG00020037170***
rs405136354	17	59,793,006	Intergenic	0.43	0.0025	2.31 × 10^−04^	*ENSOARG00020027877*, *ENSOARG00020027578*, *ENSOARG00020028857*,*ENSOARG00020030413*, *ENSOARG00020030475*, *ENSOARG00020033868*, *ENSOARG00020037170*, and *ENSOARG00020040097*
rs416601769	18	41,773,186	Intron	0.39	0.0027	4.86 × 10^−05^	***NPAS3***
s46406.1	19	60,358,663	Intron	0.25	−0.0021	5.86 × 10^−04^	*CHCHD6*, *TXNRD3*, *CHST13*, ***UROC1***, *SLC41A3*, *ALDH1L1*, *ZXDC*, *C19H3orf22*, *ENSOARG00020027839*, and *ENSOARG00020035969*

^a^ The SNP located within genes had the genes highlighted in bold.

**TABLE 7:** SNPs associated with the total number of lambs weaned (TNW) over ewe lifetime in U.S. Katahdin sheep, including chromosome (OAR) position, genomic region, MAF, and p-value of the association test.

SNP	OAR	Position (bp)	Region^a^	MAF	SNP effect	P-value	Flanking gene (±100 kb)
rs410798226	1	32,657,306	Intergenic	0.33	0.0024	2.16 × 10^−04^	
rs425311695	1	66,241,221	Intron	0.48	0.0028	4.01 × 10^−05^	*KYAT3*, *LOC101119517*, *LOC101119773*, *LOC101120395*, *ENSOARG00020028768*, *ENSOARG00020029840*, and ***ENSOARG00020006716***
rs426160726	2	155,017,143	Intergenic	0.26	0.0023	6.36 × 10^−05^	*GALNT13*, *ENSOARG00020036565*, and *ENSOARG00020026958*
rs419670964	3	76,250,495	Intergenic	0.43	−0.0025	2.42 × 10^−04^	
rs399310772	4	12,222,871	Intron	0.39	−0.0025	1.70 × 10^−04^	*BET1*, ***ENSOARG00020028395***, and ***ENSOARG00020033537***
rs411035513	6	17,589,516	Intron	0.22	−0.0020	2.11 × 10^−04^	*LEF1*, ***HADH***, *LOC101120834*, *SGMS2*, *ENSOARG00020029134*, and *ENSOARG00020029642*
rs419109423	10	73,335,461	Intron	0.25	−0.0020	4.70 × 10^−04^	***ENSOARG00020034820***
rs418344710	10	79,889,727	Intergenic	0.15	−0.0018	4.51 × 10^−04^	
rs400995964	13	7,867,793	Intergenic	0.16	−0.0019	2.93 × 10^−04^	*ENSOARG00020025339*
rs399288440	14	65,830,741	Intergenic	0.20	0.0016	5.54 × 10^−04^	*ZSCAN4*, *ENSOARG00020027141*, *LOC101110699*, *LOC101117358*, *LOC105601916*, *LOC101117613*, *LOC101110440*, and *ENSOARG00020008968*
s09727.1	17	2,089,989	Intron	0.13	−0.0016	2.46 × 10^−04^	***MAP9***, *ENSOARG00020033091*, and *ENSOARG00020036218*
rs418797399	17	58,814,585	Intron	0.48	−0.0022	5.88 × 10^−04^	***MED13L***
rs401471684	17	59,126,237	Intergenic	0.45	−0.0022	5.68 × 10^−04^	*LOC132658005*
rs405696543	17	59,616,349	Intergenic	0.45	0.0026	5.38 × 10^−05^	*ENSOARG00020028857*, *ENSOARG00020030475*, and *ENSOARG00020037170*
rs415377431	17	59,646,371	Intergenic	0.46	0.0025	7.00 × 10^−05^	*ENSOARG00020027578*, *ENSOARG00020028857*, *ENSOARG00020030475*, and *ENSOARG00020037170*
rs421430100	17	59,708,716	Intron	0.41	0.0025	6.44 × 10^−05^	*ENSOARG00020027578*, *ENSOARG00020028857*, ***ENSOARG00020030475***, and ***ENSOARG00020037170***
rs405136354	17	59,793,006	Intergenic	0.43	0.0023	4.42 × 10^−04^	*ENSOARG00020027877*, *ENSOARG00020027578*, *ENSOARG00020028857*, *ENSOARG00020030413*, *ENSOARG00020030475*, *ENSOARG00020033868*, *ENSOARG00020037170*, and *ENSOARG00020040097*

^a^ The SNP located within genes had the genes highlighted in bold.

**TABLE 8:** SNPs associated with total lamb birth weight (TLB) over ewe lifetime in U.S. Katahdin sheep, including chromosome (OAR) position, genomic region, MAF, and p-value of the association test.

SNP	OAR	Position (bp)	Region^a^	MAF	SNP effect	P-value	Candidate gene
rs425311695	1	66,241,221	Intron	0.48	0.0118	2.16 × 10^−04^	*KYAT3*, *LOC101119517*, *LOC101119773*, *LOC101120395*, *ENSOARG00020028768*, *ENSOARG00020029840*, and ***ENSOARG00020006716***
rs400022476	2	80,179,672	Intergenic	0.09	0.0067	3.05 × 10^−05^	
rs426160726	2	155,017,143	Intergenic	0.26	0.0096	2.48 × 10^−04^	*GALNT13*, *ENSOARG00020036565*, and *ENSOARG00020026958*
rs424868378	4	65,051,183	Intron	0.05	−0.0047	4.15 × 10^−04^	***BBS9***
rs405051279	9	39,147,793	Intron	0.36	0.0111	1.34 × 10^−04^	*RAB2A* and ***ENSOARG00020026533***
rs421517362	11	51,584,156	Intron	0.09	0.0055	3.82 × 10^−04^	*RNF213*, ***ENDOV***, *NPTX1*, *RPTOR*, *ENSOARG00020036732*, and *ENSOARG00020039970*
rs407234061	11	61,900,883	Intron	0.12	0.0075	5.37 × 10^−04^	***PRKCA*** and *APOH*
rs423116951	17	7,617,104	Intron	0.22	−0.0103	1.82 × 10^−04^	***ENSOARG00020021581*** and *ENSOARG00020039197*
rs416601769	18	41,773,186	Intron	0.39	0.0102	6.81 × 10^−04^	***NPAS3***
rs398978001	19	17,380,507	Intron	0.42	−0.0109	5.73 × 10^−04^	***SRGAP3***
rs405740728	22	35,912,532	Intergenic	0.11	−0.0069	5.66 × 10^−04^	*ATRNL1*, *GFRA1*, *ENSOARG00020035199*, and *ENSOARG00020040782*

^a^ The SNP located within genes had the genes highlighted in bold.

**TABLE 9:** SNPs associated with total lamb weight at weaning over ewe lifetime (TLW) and TLW divided by the ewe weight adjusted for 120 days (TLWadj) in U.S. Katahdin sheep, including chromosome (OAR) position, genomic region, MAF, and p-value of the association test.

Trait	SNP	OAR	Position (bp)	Region^a^	MAF	SNP effect	P-value	Candidate gene
TLW	rs430643857	3	193,322,988	Intron	0.08	−0.0181	1.73 × 10^−04^	*C2CD5*, *LOC101115113*, and ***ENSOARG00000020155***
TLW	rs409206327	3	211,102,082	upstream gene	0.07	−0.0182	2.15 × 10^−04^	***ENSOARG00000009243***, *KCNA1*, and *KCNA6*
TLW	rs400704482	6	72,565,414	Intron	0.08	−0.0195	3.24 × 10^−04^	*CRACD*, *AASDH*, *PPAT*, *SRP72*, *SPMAP2L*, *PAICS*, *ENSOARG00020029379*, *ARL9*, and ***ENSOARG00020027525***
TLW	rs426699276	14	4.350.042	Intron	0.40	−0.0313	5.09 × 10^−04^	
TLW	rs422018547	14	51,925,962	Intron	0.21	−0.0263	6.30 × 10^−04^	*ZNF283*, *ZNF404*, *LOC121816527*, *ZNF226, ZNF227*, ***ZNF234***, *ZNF235*, *ZNF45*, *ENSOARG00020028648*, and *ENSOARG00020030111*
TLW	rs412856165	15	49,408,866	Intergenic	0.32	−0.0307	4.42 × 10^−04^	*LOC101120356*, *TRIM68*, *LOC101113881*, *LOC101114136, ENSOARG00020011111*, and *ENSOARG000200302417431*
TLW	rs428977023	15	49,409,119	Intergenic	0.32	−0.0308	4.18 × 10^−04^	*LOC101120356*, *TRIM68*, *LOC101113881*, *LOC101114136*, *ENSOARG00020011111*, and *ENSOARG00020030241*
TLW	rs427018792	18	39,616,062	Intron	0.36	−0.0375	5.09 × 10^−04^	*HECTD1*, *HEATR5A*, *ENSOARG00020026939*, *ENSOARG00020033738*, and ***ENSOARG00000006260***
TLW	rs410487911	20	19,026,288	Intergenic	0.41	−0.0371	7.09 × 10^−05^	*CLIC5*
TLW	rs400951839	25	22,652,820	Intron	0.33	−0.0354	9.77 × 10^−05^	***CTNNA3*** and *LRRTM3*
TLW	rs161572072	25	23,833,675	Intron	0.22	−0.0304	1.63 × 10^−04^	*HERC4,* ***MYPN*** *,* and *ATOH7*
TLWadj	rs405843169	2	162,512,999	Intergenic	0.38	−0.0009	1.04 × 10^−04^	*ENSOARG00020033540*
TLWadj	rs422901910	2	220,591,671	Intron	0.20	−0.0007	2.43 × 10^−04^	*USP37*, *CNOT9*, ***PLCD4***, *ZNF142*, *BCS1L*, *RNF25*, *STK36*, and *TTLL4*
TLWadj	rs401853073	3	185,651,418	Intron	0.45	−0.0009	1.43 × 10^−04^	***TMTC1***, *OVCH1*, and *ENSOARG00020035674*
TLWadj	rs405855191	4	94,129,301	Intron	0.40	0.0009	1.35 × 10^−04^	*RBM28*, *LEP*, *ENSOARG00020027873*, ***ENSOARG00020029720***, ***ENSOARG00020030512***, *ENSOARG00020032751*, *ENSOARG00020035180*, *ENSOARG00020036546*, *ENSOARG00020037327*, ***ENSOARG00020040526***, *ENSOARG00020005028*
TLWadj	rs413623278	7	100,602,358	Intron	0.43	0.0009	3.75 × 10^−04^	***RPS6KA5***
TLWadj	rs428996958	9	33,816,660	Intergenic	0.31	−0.0008	3.90 × 10^−04^	
TLWadj	rs427207318	15	70,280,954	Intron	0.18	0.0007	2.08 × 10^−04^	***LRRC4C***
TLWadj	rs427018792	18	39,616,062	Intron	0.36	−0.0008	6.98 × 10^−04^	***ENSOARG00000006260***, *HECTD1*, *HEATR5A*, *ENSOARG00020026939*, and *ENSOARG00020033738*
TLWadj	rs405495781	22	24,041,507	Intron	0.43	0.0008	7.45 × 10^−04^	***SH3PXD2A***, *LOC101113375*, and *STN1*
TLWadj	rs400951839	25	22,652,820	Intron	0.33	−0.0009	8.74 × 10^−05^	***CTNNA3*** and *LRRTM3*
TLWadj	rs412008600	26	35,321,163	Intergenic	0.44	−0.0008	8.70 × 10^−04^	*ZMAT4*
TLWadj	rs426713590	26	36,979,940	Intron	0.29	−0.0008	6.81 × 10^−04^	*RNF170*, ***HOOK3***, *FNTA*, *THAP1*, and *ENSOARG00020040137*

^a^ The SNP located within genes had the genes highlighted in bold.

The significant associations involved 86 unique SNPs as 24 of them were associated with two or more longevity-related traits. For instance, the variants rs399310772 on OAR4:12,222,871, rs407234061 on OAR11:61,900,883, and rs400995964 on OAR13:7,867,793 were associated with five traits. The intron variant rs399310772 is located in the genes *ENSOARG00020028395* and *ENSOARG00020033537*, which encode long non-coding RNA (lncRNA) and are close to the protein-coding gene *BET1* (Bet1 Golgi vesicular membrane trafficking protein), while the SNP rs400995964 is close to the novel protein-coding gene *ENSOARG00020025339*. Both SNPs were associated with ALL, LPL, TNL, TNB, and TNW. The SNP rs407234061, an A>G change in intron 1 of the *PRKCA* (protein kinase C alpha), was found to be associated with ALL, LPL, TNL, TNB, and TLB.

The 86 variants associated with the longevity-related traits are distributed in different genomic regions, including intergenic (31 SNP), intron (50 SNP), exon (3 SNP), upstream gene (1 SNP), and downstream gene (1 SNP) variants. The MAF of these variants ranged from 0.05 to 0.48 in the current Katahdin population. The missense SNP rs423900190 on OAR6:85,982,278 was associated with both ALL (Table 3) and TNB (Table 6). This is a T>C variant in exon 6 of the *SULT1B1* (sulfotransferase family 1B member 1) gene, which results in an amino acid change from isoleucine to valine at position 136. The variants (Table 3) rs160278050 (OAR7:10,694,207) and rs160278082 (OAR7:10,695,388), also associated with ALL, are T>G and A>G substitutions, respectively, located in exon 2 of the *CMYA5* (cardiomyopathy-associated 5) gene. These variants result in amino acid changes from valine to glycine (at position 2,654) and lysine to glutamic acid (at position acid 3,048).

### 3.2 Functional annotation analyses

Genome windows of 100 Kbp downstream and 100 Kbp upstream of each significant SNP were selected for functional analyses, and 272 genes were found. Of these, 203 are protein- and 69 RNA-coding genes (Supplementary File S1). Of the protein-coding genes, 174 have a biological function known, and 29 are novel genes. Of the RNA-coding genes, 52 encode long non-coding RNAs (lncRNAs), eight encode small nucleolar RNAs (snoRNAs), six encode small nuclear RNAs (snRNAs), and three encode microRNAs (miRNAs).

A list of 272 Ensembl gene identification codes was submitted to the DAVID tool, and 183 genes were retrieved. Three genes (*ADCY5*, *PRKAB1*, and *RPTOR*) are key components in the longevity-regulating pathway. Twenty-three genes (*HACD2*, *ATP6V1C1*, *ST3GAL4*, *LOC101119640*, *ADCY5*, *ALDH1L1*, *CKB*, *LOC101120834*, *LOC101121036*, *LOC101113375*, *LOC105608607*, *FECH*, *HADH*, *KYAT3*, *KMT5C*, *NOS1*, *PPAT*, *PAICS*, *GALNT13*, *SGMS2*, *LOC101120331*, *TMEM86B*, *and UROC1*) are associated with metabolic pathways. Seven genes (*ADCY5*, *CACNG6*, *CACNG7*, *CACNG8*, *PRKAB1*, *PRKCA*, and *PRKCG*) are associated with the oxytocin signaling pathway. Some genes play important roles in health, such as *ADCY5*, *CACNG6*, *CACNG7*, *CACNG8*, *CTNNA3*, *ITGAV*, *LEF1*, *COX6B2*, *MYL2*, *NOS1*, *PRKAB1*, *PRKCA*, *RPS6KA5*, and *TNNI3*, which are components of cardiac pathways, such as hypertrophic cardiomyopathy, dilated cardiomyopathy, arrhythmogenic right ventricular cardiomyopathy, adrenergic signaling in cardiomyocytes, and cardiac muscle contraction. Other genes are related to the immune system (*LOC101110699*, *LOC101110440*, *LOC105601916*, *ZNF227*, *ZNF235*, *ZNF283*, *ZNF404*, *LOC101117358*, *LOC101117613*, *CTNNA3*, *MYL2*, *PRKCA*, and *PRKCG*) and are components of pathways such as the herpes simplex virus 1 infection pathway and leukocyte transendothelial migration. There were also genes related to signaling pathways such as *ITGAV*, *MED13L*, *PRKCA*, and *PRKCG* (thyroid hormone signaling pathway); *RAB2A*, *LEP*, *PRKAB1*, and *RPTOR* (AMPK signaling pathway); and *CACNG6*, *CACNG7*, *CACNG8*, *PRKCA*, *PRKCG*, and *RPS6KA5* (MAPK signaling pathway). The genes *LOC101119773*, *LOC101119517*, and *LOC101120395* are members of the guanylate-binding protein family and are related to immunity and innate immunity biological processes.

**FIGURE 4:**
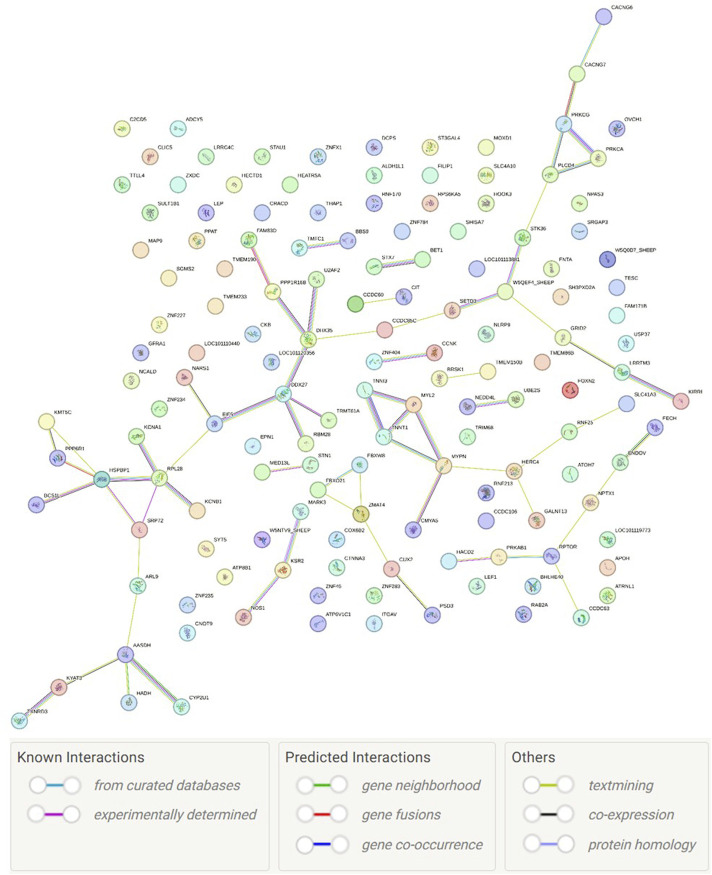
Protein–protein network interaction and color legends.

Although the genes are involved in many pathways, only six enriched pathways were found, namely, hypertrophic cardiomyopathy pathway (*P* = 0.00002), dilated cardiomyopathy pathway (*P* = 0.000028), arrhythmogenic right ventricular cardiomyopathy (*P* = 0.000084), adrenergic signaling in cardiomyocytes (*P* = 0.00039), oxytocin signaling pathway (*P* = 0.0020), and cardiac muscle contraction (*P* = 0.0023). A UniProt term called transferase (KW-0808; *P* = 0.0032; with 17 genes: *BRSK1*, *HECTD1*, *NEDD4L*, *ST3GAL4*, *LOC101119640*, *CIT*, *LOC105608607*, *FNTA*, *KSR2*, *KYAT3*, *KMT5C*, *PPAT*, *GALNT13*, *PRKCA*, *PRKCG*, *RPS6KA5*, *RNF170*, *STK36*, *SGMS2*, *LOC101120084*, *LOC101120331*, *TRMT61A*, *TENT2*, and *UBE2S*) was found to be enriched. Finally, the protein–protein interaction network (Figure 4) had significantly more interactions than expected (*P* = 0.0371).

The search for QTLs in the genome windows ±100 Kbp also found interesting results (Table 10). For instance, there were QTLs from OAR10:26,866,363 to OAR10:26,995,420 previously associated with horn type and horn circumference. QTLs for health-related traits such as pleurisy, mean corpuscular hemoglobin concentration, and *Haemonchus contortus* resistance have been previously reported on OAR2:136,270,247–136,270,251 bp, OAR6:17,589,514–17,589,518 bp, OAR8:2,217,907–2,217,911 bp, OAR10:73,366,801–73,366,805 bp, and OAR17:55,671,815–59,533,215 bp. For lactation-related traits such as milk, fat, and protein yields, there were QTLs located on OAR3:63664566–63664570 bp, OAR4:94162840–94162844 bp, OAR13:67636466–67769899 bp, OAR17:58854886–58854890 bp, and on OAR21:27132598–27132602 bp, while QTLs for growth-related traits such as average daily gain and body weight were identified on OAR4:94162840–94162844 bp and OAR14:62350077–62350081 bp. The enrichment analyses found an enriched QTL for *H. contortus* resistance on OAR17 (*P* = 0.0007) and another on OAR6 for mean corpuscular hemoglobin concentration (*P* = 0.0076).

**TABLE 10:** Quantitative trait loci (QTL) previously mapped close to the SNPs associated with longevity-related traits in the U.S. Katahdin population.

OAR	Confidence interval		QTL_ID	Trait	PUBMED_ID	SNP Flanking
	Start (bp)	End (bp)				
2	136,270,247	136,270,251	170,705	Pleurisy	30099550	rs415671617
2	136,270,247	136,270,251	170,705	Pleurisy	30099550	rs415671617
3	63,664,566	63,664,570	169,218	Milk protein yield	32510640	rs403002296
3	63,664,566	63,664,570	169,217	Milk yield	32510640	rs403002296
4	94,162,840	94,162,844	193,115	Milk yield	31979402	rs420693815
4	94,162,840	94,162,844	193,116	Milk fat percentage	31979402	rs420693815
4	94,162,840	94,162,844	193,121	Body weight	31979402	rs420693815
4	94,162,840	94,162,844	193,122	Average daily gain	31979402	rs420693815
6	17,589,514	17,589,518	213,331	Mean corpuscular hemoglobin concentration	24023702	rs411035513
8	2,217,907	2,217,911	259,456	*Haemonchus contortus* resistance	36015059	rs402371066
10	26,866,363	26,866,367	161,485	Horn type	21651634	rs419996970
10	26,866,363	26,866,367	161,411	Horn type	21651634	rs419996970
10	26,922,986	26,922,990	161,412	Horn type	21651634	rs405467063
10	26,922,986	26,922,990	161,486	Horn type	21651634	rs405467063
10	26,922,986	26,922,990	161,399	Horn circumference	21651634	rs405467063
10	26,954,784	26,954,788	161,413	Horn type	21651634	rs416770048
10	26,954,784	26,954,788	161,487	Horn type	21651634	rs416770048
10	26,954,784	26,954,788	161,400	Horn circumference	21651634	rs416770048
10	26,995,416	26,995,420	161,414	Horn type	21651634	rs408429296
10	26,995,416	26,995,420	161,488	Horn type	21651634	rs408429296
10	73,366,801	73,366,805	213,288	Mean corpuscular hemoglobin content	24023702	rs401697603
13	67,636,466	67,636,470	169,542	Milk fat yield	32510640	rs428538826
13	67,769,895	67,769,899	169,273	Milk fat yield	32510640	rs407472479
14	62,350,077	62,350,081	277,203	Average daily gain	36980850	rs406902335
17	55,671,815	55,671,819	259,462	*Haemonchus contortus* resistance	36015059	rs399621490
17	55,717,058	55,717,062	259,463	*Haemonchus contortus* resistance	36015059	rs410780866
17	55,717,058	55,717,062	259,463	*Haemonchus contortus* resistance	36015059	rs410780866
17	58,854,886	58,854,890	57,700	Milk fat percentage	23094085	rs411845108
17	58,854,886	58,854,890	57,700	Milk fat percentage	23094085	rs411845108
17	59,533,211	59,533,215	259,461	*Haemonchus contortus* resistance	36015059	rs425080766
17	59,533,211	59,533,215	259,461	*Haemonchus contortus* resistance	36015059	rs425080766
21	27,132,598	27,132,602	170,221	Milk yield	30983012	rs398340969
21	27,132,598	27,132,602	170,231	Milk protein yield	30983012	rs398340969

## 4 Discussion

### 4.1 Association analyses

Ewe longevity has a major economic impact (Krupová et al., 2009; Wolfová et al., 2009b; 2009a), but longevity-related traits are not often directly included in sheep breeding schemes. This occurs because traits such as ALL and LPL often have low heritability estimates (Riggio et al., 2009; Milerski et al., 2018; Pelmuş et al., 2020; Medrado et al., 2021) and are expressed late in an ewe’s life. These issues allow the additional use of genomics for longevity traits to have a greater impact in breeding programs. This was the first study to perform GWASs for longevity-related traits in the U.S. Katahdin sheep. Moreover, as far as we know, only one recent previous study reported GWASs for longevity traits in sheep (Smitchger et al., 2024), which evaluated ALL, TNL, TNB, TNW, and TLW recorded in 1,130 ewes from four U.S. sheep breeds (Rambouillet, Polypay, Suffolk, and Columbia). The present study performed GWAS analyses using a breed with a larger genotypic dataset and additional traits such as LPL, TLB, and TLWadj. Moreover, the previous study analyzed data from a single production system, while in the current study, a wide array of production systems and flock locations were analyzed. A greater number of genome-wide associations (n = 25) was reported than that in the present study (Smitchger et al., 2024). Moreover, the genomic regions reported in the previous study did not overlap the positions reported in the current study. Differences between GWAS results from different studies and populations may be a consequence of the variability (genotypic and phenotypic) observed for longevity-related traits across breeds (Annett et al., 2011; Hanna et al., 2023), trait definition, statistical models used, data editing and quality control procedures, and stringency of multiple testing correction. Moreover, longevity indicators show a complex polygenic control, where each locus has a small effect on the total variability of the trait, which makes it more difficult to identify the same locus explaining variance in longevity traits across breeds and even in different datasets from the same breed.

We identified 24 markers associated with two or more traits, which is strong evidence that a high genetic correlation between these traits exists. Genetic correlations greater than 0.8 between these eight longevity traits have been observed (Pinto et al., 2025b). In a multi-breed U.S. population, Pearson correlations ranging from 0.82 to 0.97 between ALL, TNL, TNB, TNW, and TLW were reported (Smitchger et al., 2024). In Spanish Churra ewes, genetic correlations of 0.96 (ALL x LPL), 0.97 (ALL x TNL), and 0.96 (LPL x TNL) were also reported (El-Saied et al., 2005). In Dorper sheep, the genetic correlation between TNB and TNW was 0.85 (Zishiri et al., 2013). Therefore, these longevity indicators are likely to have a similar genetic architecture.

Ewe longevity depends on several factors (McLaren et al., 2020). In general, highly productive ewes have longer lifespans than low-producing ewes because this second group is voluntarily culled earlier. However, productive ewes may die, or breeders may need to cull highly productive ewes due to disease or reproductive issues, which are involuntary culling reasons. Unfortunately, the culling reasons for Katahdin ewes were not recorded within the NSIP. However, previous sheep studies have reported disease and reproductive issues as some of the main causes of involuntary culling (McLaren et al., 2020; Ridler et al., 2024). Furthermore, Katahdin breeders tend to use estimated breeding values for the number of lambs born and the number of lambs weaned to select replacement animals (Notter and Lewis, 2018), which is expected to impact ewe lifetime, as low genetic merit ewes for these traits have increased chances of being culled earlier compared to high genetic merit ewes. Therefore, it is expected that genes related to factors such as reproduction, diseases, immunity, and growth performance will be identified when GWASs are performed for ewe longevity indicators.

### 4.2 Reproduction-related genes

In U.S. Katahdin sheep, the number of lambs born and the number of weaned are two key selection criteria used to increase ewe reproductive performance (Notter and Lewis, 2018). Thus, high prolificacy ewes have increased chances of being retained in the flock for a longer time than less prolific ewes. Many genes have been reported as candidates for litter size in various sheep breeds (Abdoli et al., 2016) and some of these genes were found close to the SNPs associated with longevity. For instance, the SNP rs407234061 on OAR11:61,900,883 was associated with five traits (ALL, LPL, TNL, TNB, and TLB), and close to this variant is the *APOH* (apolipoprotein H) gene, which is involved in lipoprotein metabolism. Previous studies reported a role of this gene in both women (Miettinen et al., 2001) and cow (Minozzi et al., 2013) fertility. Furthermore, *APOH* has been reported as a candidate gene for prolificacy in sheep (Wang et al., 2024). The SNP rs428707491 on OAR14:62,610,160 was associated with ALL and LPL, and it is close to the *NLRP9* (NLR family pyrin domain containing 9) gene, which is a member of a family that encodes intracellular proteins with critical roles in inflammatory response, early mammalian embryogenesis, and reproduction (Van Gorp et al., 2014). *NLRP9* was suggested as a candidate gene for prolificacy in both sheep (Zhang et al., 2020) and pigs (Zhang Y. et al., 2023). The variants rs421227438 on OAR18:65,806,713, which are also associated with ALL, are close to the *CKB* (creatine kinase B) gene. A previous study reported that *CKB* may be associated with a better ability to defend against oxidative stress, resulting in good-quality embryos (Lee et al., 2010). Moreover, a homozygous region close to the *CKB* gene was associated with litter size in Hu sheep (Tao et al., 2021). The variant rs405495781 on OAR22:24,041,507 was associated with TLWadj, and close to this position was found the *SH3PXD2A* (SH3 And PX Domains 2A) gene, which acts as an organizer protein that facilitates NOX1-or NOX3-dependent reactive oxygen species (ROS) generation and ROS localization. *SH3PXD2A* was previously reported as a candidate gene for litter size in pigs (Mo et al., 2022). On OAR25:23,833,675 was found the SNP rs161572072, which was found to be associated with TLW, and close to this position is the *HERC4* (HECT and RLD domain containing E3 ubiquitin protein ligase 4) gene. *HERC4* was overexpressed in ovary of the hyper prolific Small Tail Han sheep compared to Dorset sheep (Miao et al., 2016), suggesting a potential role in sheep prolificacy.

Our study also found other genes that, although do not have evidence of a direct relationship with prolificacy, were reported as candidates for enhancing mammalian fertility. In several mammalian species, aging promotes a continuous decline in the ovarian follicle pool until reaching the end of the female reproductive stage (Winkler and Goncalves, 2023), a physiological process called menopause in humans. The SNP rs426160726 on OAR2:155,017,143 was found to be associated with TNL, TNB, TNW, and TLB, and close to this variant is the *GALNT13* (polypeptide N-acetylgalactosaminyltransferase 13) gene, which is involved in the metabolism of proteins and was reported as a candidate gene for age at menopause in women (Lunetta et al., 2007). Another SNP, rs420711470 on OAR14:62,954,922, was associated with ALL and LPL, and close to this variant was found the *TMEM150B* (transmembrane protein 150B) gene. *TMEM150B* increases cell survival by triggering autophagy when cells are in a stressful environment (Mrschtik and Ryan, 2016). Autophagy may be a cell survival mechanism to maintain the provision of female germ cells before establishing primordial follicle pools in the ovary (Gawriluk et al., 2011). *BRSK1* (BR serine/threonine kinase 1) is another gene close to the SNP rs420711470, which affects the release of the gonadotropin-releasing hormone from the hypothalamic–pituitary–ovarian axis (Qin et al., 2012). Variants in both *TMEM150B* and *BRSK1* genes were associated with age at menopause and length of reproductive lifespan in women (He et al., 2009; Stolk et al., 2009; Chen et al., 2012). Moreover, *BRSK1* was reported as a candidate gene for conceptus elongation in sheep (Brooks et al., 2015).

Female reproductive activity depends on a complex physiological process that involves multiple hormones, and some genes identified in the current study are involved with the synthesis and release of reproductive hormones. The SNP rs423900190 on OAR6:85,982,278 was associated with ALL and TNB, and close to this variant were found the genes *SULT1B1* (sulfotransferase 1B1) and *SULT1E1* (sulfotransferase 1E1), two sulfotransferases involved in the metabolism of endogenous compounds such as thyroid and steroid hormones, including estradiol inactivation (Yi et al., 2021), which play a key role in mammalian pregnancy (Bairagi et al., 2018). In another region of OAR6 was found the variant rs411035513 (OAR6:17,589,516), which was found to be associated with TNW, which is close to the *LEF1* (lymphoid enhancer binding factor 1) gene. *LEF1* was required for the formation of endometrial glands in the mouse uterus, and its expression varied cyclically with the mouse estrus cycle (Shelton et al., 2012). Moreover, *LEF1* was reported as a candidate gene for fertility-related traits in Hu sheep by regulating the function of the reproductive axis (Yao et al., 2022). On OAR18:65,806,713 was found the SNP rs421227438, which was found to be associated with ALL, which is close to the *EIF5* (eukaryotic translation initiation factor 5) gene. *EIF5* may increase circRNAs to promote the expression of some other genes related to hormone activities such as GnRH, with a potential impact on sheep reproduction (Zhang et al., 2019) and goat puberty precocity (Liu Y. et al., 2023).

We also found some genes that may affect the early stages of pregnancy. On OAR2:122,777,052 was found the SNP rs402224636, which was found to be associated with ALL and TNL, and close to this SNP is the *ITGAV* (integrin subunit alpha V) gene, which encodes essential cell adhesion molecules that improve endometrial receptivity in pregnancy success (Ai et al., 2021). Moreover, *ITGAV* promotes endoderm differentiation (Brafman et al., 2013), which is central to embryonic development. In a different region of OAR2 is the SNP rs413377527 (OAR2:148,006,184) associated with LPL. Close to this SNP is the *SLC4A10* (solute carrier family 4 member 10) gene, a member of a family of sodium/bicarbonate cotransporters, which assists in the regulation of intracellular pH with impacts on mammalian reproduction, including early embryo development (Liu et al., 2012). *SLC4A10* was also reported as a candidate gene for TLW in South African Merino (Snyman et al., 2020). Therefore, *SLC4A10* may also play a potential role in ewe maternal ability, increasing offspring weight, with a positive effect on ewe longevity. Close to the SNP rs411035513 (on OAR6:17,589,516), the *HADH* (hydroxyacyl-CoA dehydrogenase) gene was found, which may play a role in inhibiting the proliferation of mouse embryonic fibroblasts (Xia et al., 2022). On OAR13:77,378,009 was found the SNP rs416157010, which was found to be associated with TNB, and in this region is the *ZNFX1* (zinc finger NFX1-type containing 1) gene, a component of the regulation of the telomerase pathway, and is required against some bacterial infections. Although its role in reproduction is unclear, *ZNFX1* had increased expression in pregnant compared with non-pregnant cows (Forde et al., 2012), suggesting that genes involved in the immune response may affect uterine receptivity to implantation. On OAR14:65,830,741 was found the SNP rs399288440, which was found to be associated with ALL, TNL, TNB, and TNW, and close to this SNP, another zinc finger gene, *ZSCAN4* (zinc finger and SCAN domain containing 4), was found. *ZSCAN4* is a member of the pre-implantation embryo pathway, which is essential for the early embryonic development of bovine (Liu B. et al., 2023) and swine (Zhang et al., 2022). On OAR14:62,610,160 was found the SNP rs428707491, which was found to be associated with ALL and LPL, and close to this region was found the *EPN1* (Epsin 1) gene, which is a component of the membrane trafficking pathway and was found to be differentially expressed in uterine epithelial tissue during the peri-implantation period of pregnancy in sheep (Brooks et al., 2016). On OAR17:57,981,108 was found the SNP rs427138541, which was associated with LPL. Close to this SNP is the *FBXW8* (F-box and WD repeat domain containing 8) gene, which plays an important role in the mid-to-late stage of placenta development in mice (Kinterová et al., 2022), and it has been suggested as a candidate gene for goat fertility (Sun et al., 2022). Also close to the SNP rs427138541 is the *NOS1* (nitric oxide synthase 1) gene, a member of the enzyme family that synthesizes nitric oxide; this gene plays several roles in animal reproduction, including spermatogenesis, follicle development, steroidogenesis, and ovulation (Zhang W. et al., 2023). Moreover, *NOS1* was identified as a candidate gene for heat tolerance in sheep (Li et al., 2019), and heat stress can reduce ewe reproductive performance (Van Wettere et al., 2021). On OAR21:27,230,215 was found the SNP rs408858996, which was found to be associated with TNL, and in this region is the *ST3GAL4* (ST3 beta-galactoside alpha-2,3-sialyltransferase 4) gene, which had lower expression in the cervical mucus of Suffolk ewes compared with high-fertility sheep breeds (Abril-Parreño et al., 2022), suggesting a potential effect on ewe fertility. On OAR22:35,912,532 was found the SNP rs405740728, which was found to be associated with TLB, and close to this region is the *GFRA1* (GDNF family receptor alpha 1) gene, a receptor of glial cell-line-derived neurotrophic factor, which promotes primordial follicle development and mediates autocrine and paracrine cell–cell interactions required during folliculogenesis (Dole et al., 2008). *GFRA1* has been reported as an important gene in bovine oocyte maturation and early embryo development (Kamalludin et al., 2018; Wang et al., 2018). The current study also identified 52 lncRNA-encoding genes on different chromosomes. A differential expression study in the ovaries of low- and high-fecundity Hanper sheep identified the lncRNA Xist (loc101112291) and Gtl2 (loc101123329) genes to be highly expressed, which suggests an effect on the regulation of follicular development by mediating methylation processes (Liu et al., 2021). Moreover, lncRNAs may be a key regulator of the functions in sheep oviductal tissue (Chen et al., 2024).

Some genes that can indirectly affect ewe reproductive performance were also found. For instance, on OAR1:188,636,771 was found SNP rs161707914, which was found to be associated with TNB, and close to this SNP is the *ADCY5* (adenylate cyclase 5) gene, which encodes an enzyme that catalyzes the conversion of ATP to cAMP, and plays crucial roles in normal biological functions (e.g., lipolysis, gluconeogenesis, and respiration) and pathophysiological states (e.g., diabetes and obesity) (Vatner et al., 2013). *ADCY5* has been suggested as a candidate gene for longevity in mammalian species (Yan et al., 2007; Cash et al., 2014) and for fertility-related traits in multiple livestock species, including sheep (Martinez-Royo et al., 2017; Du et al., 2022), cattle (Li et al., 2021), pigs (Sun et al., 2023), and ducks (Bello et al., 2021). On OAR4:65,051,183 was found the SNP rs424868378, which was found to be associated with TLB, and close to this SNP is the *BBS9* (Bardet–Biedl syndrome 9) gene, a member of a family of genes that cause Bardet–Biedl syndrome, which is characterized by various hypogonadism or genitourinary abnormalities such as a malformed uterus, hydrometrocolpos, and vaginal atresia, resulting in irregular menstrual cycle and polycystic ovaries (Melluso et al., 2023). In pigs, a recessive lethal deletion in *BBS9* was assumed to cause fetal lethality in mutant homozygotes, which has antagonistic pleiotropic effects on fertility and growth (Derks and Steensma, 2021).

### 4.3 Health-related genes

Diseases are one of the main causes of involuntary culling in many ruminant species, including dairy and beef cattle (Aleri et al., 2021; Souza et al., 2023), goats (Malher et al., 2001; Davachi, 2019), and sheep (McLaren et al., 2020). Haemonchosis is a critical infectious disease in sheep, which is caused by the gastrointestinal nematode *H. contortus* (Flay et al., 2022). We found significant SNPs on OAR8:2,269,243 (rs409333251) and OAR17 (rs405696543—OAR17:59,616,349, rs419277881—OAR17:55,794,456, and rs424778250—OAR17:55,644,362) close to regions where QTLs were reported for *H*. *contortus* resistance (Niciura et al., 2022). Moreover, we also found SNPs on OAR6:17,589,516 (rs411035513) and OAR10:73,335,461 (rs419109423), where QTLs for mean corpuscular hemoglobin concentration/content (MCHC) were reported (Gonzalez et al., 2013). MCHC is an indicator of anemia and can be used to identify sheep that are more resistant to gastrointestinal nematodes (Jiménez-Penago et al., 2021). Our study found an association between the intron variant rs411035513 in *HADH* (hydroxyacyl-CoA dehydrogenase) and TNW, while an association of this same SNP with MCHC was also reported (Gonzalez et al., 2013), suggesting a possible pleiotropic QTL in this region of OAR6. We found a variant (rs405740728) on OAR22:35,912,532 associated with TLB, and close to this position is the *ATRNL1* (attractin like 1) gene. Variation in *ATRNL1* was associated with fecal egg count in Akkaraman sheep (Arzik et al., 2022), which is an indicator of parasite resistance, but there is no QTL reported for fecal egg count in this region of OAR22 in the Sheep QTLdb (www.animalgenome.org/cgi-bin/QTLdb/OA/index). Moreover, long non-coding RNA genes may also be associated with response against parasite infections in sheep (Chitneedi et al., 2021), and the present study found 52 lncRNA genes.

Other chromosomal regions identified in the current study are not close to the previous health QTLs reported in Sheep QTLdb (www.animalgenome.org/cgi-bin/QTLdb/OA/index), but they may also be related to physiological responses to health issues. On OAR4:94,129,301 is the SNP rs405855191, which was associated with TLWadj, and close to this position was found the *LEP* (leptin) gene. This gene regulates the stem cell numbers and functions in hematopoiesis under homeostasis or stress-associated situations and pathological conditions (Trinh and Broxmeyer, 2015), and continuous blood cell production is a key response against anemia caused by *H. contortus* infection. On OAR14:62,954,922 was found the SNP rs420711470, which was found to be associated with ALL and LPL, and close to this SNP is the *IL11* (interleukin 11) gene, another gene with hematopoietic effects and potential therapeutic value in anemia conditions (Du and Williams, 1997). Moreover, the interleukin-11 signaling pathway plays a key role in maintaining adequate blood volume levels, which is especially important after sheep are infected by *H*. *contortus* (Al Kalaldeh et al., 2019). Another SNP in OAR14 is rs428707491 (OAR14:62,610,160), which was also associated with ALL and LPL, and close to this SNP is the *NLRP9* (on OAR14) gene. This gene initiates cytosolic multiprotein complexes of the innate immune system to activate an inflammatory response to intestinal infection and could also impact responses to gastrointestinal nematode infections (Mullins and Chen, 2021). The intron variant rs407234061 in the *PRKCA* gene (OAR11:61,900,883) was associated with five longevity traits, while on OAR14:62,375,192 was found the SNP rs411860439, which was found to be associated with ALL, and close to this SNP is the *PRKCG* (protein kinase C gamma) gene. These are two gene members of the large family of protein kinase C, which play key roles in hematopoietic and immune responses (Altman and Kong, 2016). In sheep, *PRKCA* was found as a differentially expressed gene during subclinical paratuberculosis infection (Purdie et al., 2019).

On OAR18:39,616,062 was found the SNP rs427018792, which was found to be associated with TLW and TLWadj, and close to this SNP is the *HECTD1* (HECT domain E3 ubiquitin protein ligase 1) gene. *HECTD1* is involved in multiple pathways such as adaptive immune system, antigen processing, ubiquitination and proteasome degradation, class I MHC-mediated antigen processing and presentation, and immune system pathways. Moreover, *HECTD1* was reported as a candidate gene for somatic cell count in Frizarta dairy sheep (Kominakis et al., 2019). On OAR23:57,717,942 is the SNP rs411106195, an intron variant in *NEDD4L* (NEDD4-like E3 ubiquitin protein ligase) gene, which was associated with LPL. *NEDD4L* is a component of several pathways related to immunity such as infectious disease and innate immune system pathways. Moreover, *NEDD4L* was suggested as a candidate gene for gastrointestinal nematode infection-related traits in cattle (Wolf et al., 2021). On OAR23:57,231,444 is the SNP rs400623485, which was associated with TNL. Close to this SNP is the *FECH* (ferrochelatase) gene, which encodes an enzyme that catalyzes the terminal step of the heme biosynthesis pathway, which is essential for many hemoproteins, including hemoglobin (Medlock and Dailey, 2022). In humans, *FECH* variants were associated with mean corpuscular hemoglobin volume and concentration, red blood cell distribution width, and mean corpuscular volume (Sakaue et al., 2021), i.e., blood traits that may play a role against *H. contortus* infection.

On OAR25:22,652,820 was found the SNP rs400951839, which was associated with TLW and TLWadj, and close to this SNP are the *CTNNA3* (Catenin Alpha 3) and *LRRTM3* (leucine-rich repeat transmembrane neuronal 3) genes, which have been associated with several brain disorders in humans according to the GeneCards database. A recent study reported the region of OAR25 close to the genes *CTNNA3* and *LRRTM3* as being prone to escape reprogramming and a potential candidate for transgenerational epigenetic inheritance, suggesting a potential genetic overlap between brain and infertility disorders (Braz et al., 2024). Our study also found SNPs close to various genes that encode zinc-finger proteins (ZNF genes), which play a known role in the development and differentiation of several tissues and are involved in the onset of several diseases (Cassandri et al., 2017).

### 4.4 Growth- and milk-related genes

Increasing the growth rate is a major selection objective in meat sheep breeds, and lamb growth depends on direct and maternal genetic components (Pech et al., 2012). Growth-related selection criteria such as birth and weaning weights are currently used in U.S. Katahdin sheep (Notter and Lewis, 2018). Thus, it is expected that ewes with low-EBV for these traits will likely be culled earlier than ewes with higher genetic merit. Our study found genomic regions close to QTL reported for growth- and milk-related traits, which may impact Katahdin lamb growth and have a consequent impact on ewe longevity. Functional annotation analyses enabled the identification of candidate genes in these regions.

The intron variant rs400216417 on OAR6:32,071,830 was associated with LPL. This variant encodes the *GRID2* (glutamate ionotropic receptor delta type subunit 2) gene, a component of intracellular calcium signaling, which plays a role in calcium homeostasis. Calcium is required for animal growth and milk production (Ni et al., 2024). The intron variant rs418797399 on OAR17:58,814,585 was associated with ALL and TNW. This variant encodes the *MED13L* (mediator complex subunit 13L) gene, a component of metabolic pathways, and was reported as a candidate gene for growth-related traits in cattle (Ma et al., 2023). The variant rs405855191 on OAR4:94,129,301 was associated with TLWadj, and close to this position was found the *LEP* gene. *LEP* is associated with energy homeostasis, neuroendocrine and immune functions, and the metabolism of glucose, lipids, and bone (Park and Ahima, 2015). In sheep, *LEP* was reported as a candidate gene associated with many traits, including carcass-related traits (Meira et al., 2018), body fat reserves (Macé et al., 2022), morphology, and body weight (Shojaei et al., 2010; Machado et al., 2020; Darwish et al., 2023). Leptin is also involved in skeletal development as it increases the abundance of the insulin-like growth factor 1 receptor (*IGF1R*) within the chondrocyte and progenitor cell populations (Reid et al., 2018). This receptor has been reported as a candidate gene for both sheep growth (Proskura et al., 2014) and longevity (Byun et al., 2012). Therefore, *LEP* gene may have an indirect effect on longevity by increasing the *IGF1R* level. On OAR21:27,230,215 was found the variant rs408858996, which was associated with TNL, and close to this variant is the *DCPS* (decapping enzyme, scavenger) gene, which has been suggested as a candidate gene for milk and protein yields in both sheep (Sutera et al., 2019) and cattle (Suchocki et al., 2016); therefore, *DCPS* may have a potential effect on animal growth by affecting milk-related traits.

### 4.5 Carcass-related genes

The improvement of carcass traits is also the target of selective schemes in meat sheep breeding programs, and at least two genes identified in the current study were also reported to be associated with carcass traits in sheep. On OAR7:10,694,207 and OAR7:10,695,388 were found two missense variants, rs160278050 and rs160278082, respectively, in exon 2 of the *CMYA5* gene, which encodes myospryn, a protein expressed predominantly in skeletal and cardiac muscles (Benson et al., 2017). Myospryn is an inhibitor of the calcineurin signaling pathway in skeletal muscle, playing an important regulatory function in muscle differentiation, fiber-type determination, hypertrophy, and muscle regeneration (Kielbasa et al., 2011). Some previous studies reported *CMYA5* as a candidate gene for carcass- and meat quality-related traits in cattle (Bruscadin et al., 2021) and pigs (Xu et al., 2011). On OAR18:62,114,682 is the SNP s37838.1, an intron variant in the *SETD3* (SET domain containing 3 actin N3 (tau)-histidine methyltransferase) gene. *SETD3* is associated with multiple other genes, regulating various biological processes such as cell cycle and apoptosis (Witecka et al., 2021). In sheep, *SETD3* was suggested as a candidate gene for carcass-related traits (Souza et al., 2022).

### 4.6 Metabolic pathways

Our study also identified more protein–protein interactions than expected (Figure 4). Protein–protein interactions mediate essentially all biological processes, especially in the disease context (Lage, 2014). These interactions indicate that many of the candidate genes found in the current study may be acting together on phenotype expression. For instance, the genes *PRKCG* and *PRKCA* are components of multiple pathways, such as GnRH secretion and gastric acid secretion, while *PRKCA* and *PLCD4* are components of the thyroid hormone and calcium signaling pathways. Physiological mechanisms associated with longevity have been more extensively studied in species such as humans and rodents since livestock animals are usually culled before reaching old age. Several genes (*HACD2*, *ATP6V1C1*, *ST3GAL4*, *LOC101119640*, *ADCY5*, *ALDH1L1*, *CKB*, *LOC101120834*, *LOC101121036*, *LOC101113375*, *LOC105608607*, *FECH*, *HADH*, *KYAT3*, *KMT5C*, *NOS1*, *PPAT*, *PAICS*, *GALNT13*, *SGMS2*, *LOC101120331*, *TMEM86B*, and *UROC1*) found in the current study are components of metabolic pathways, which are known to play a key function in feed intake (Jorge-Smeding et al., 2021). These genes can be involved in maintaining a good nutritional status of mammalians, especially based on low-calorie diets, which has been suggested as an efficient mechanism for attenuating aging (Flanagan et al., 2020), because excessive energy intake and adiposity can cause systemic inflammation (Kökten et al., 2021). Moreover, some signaling pathways associated with longevity were also found. For instance, the thyroid hormone signaling pathway may modulate lifespan in mammals as thyroid hormones are involved with activating, inhibiting, or modulating the gene expression of key regulators of metabolism, growth, and inflammation (Bowers et al., 2013). The AMPK signaling pathway may also be related to aging as AMPK activation extends the lifespan in both *Caenorhabditis elegans* and rodents (Yu et al., 2021), which may be a consequence of AMPK roles in the regulation of cellular homeostasis, resistance to stress, cell survival and growth, cell death, and autophagy. MAPK signaling is another essential pathway for many biological processes (e.g., proliferation, differentiation, apoptosis, and stress response), and MAPK enzymes may be upregulated with aging (Cano et al., 2019). Finally, three genes (*ADCY5*, *PRKAB1*, and *RPTOR*) found in the current study are components of the longevity-regulating pathway, indicating that the genomic regions close to the positions OAR1:188,636,771, OAR17:55,794,456, and OAR11:51,584,156 may be associated with ewe longevity.

The current study also found seven genes that are components of the oxytocin signaling pathway, which were enriched in our analyses. The oxytocin hormone plays a key role during the peripartum period by stimulating physiological events (e.g., myometrial contractions and prostaglandin production), which makes the exogenous application of oxytocin a veterinary practice often used to induce parturition (Marcet-Rius et al., 2023). Oxytocin also has functions in sheep lactation, such as stimulating milk ejection, sustaining mammary cells, and increasing lactation persistency (Nezamidoust et al., 2015). Oxytocin is considered an essential component to stimulate maternal behaviors, which include nest-building, reluctance to leave the nest, genital and overall licking of the newborn, nursing, and direct contact with the litter (Mota-Rojas et al., 2023). Assuming that lambing issues and reduced maternal behavior may be influencing culling of ewes, our results suggest that the oxytocin signaling pathway may be involved with Katahdin ewe longevity.

Our study found enriched pathways related to the cardiovascular system. Cardiovascular diseases are the leading cause of death in humans aged 65 years or older (North and Sinclair, 2012), indicating a relationship between the deterioration of cardiac function and aging. The health of the cardiovascular system contributes to delivering oxygenated blood to other body tissues, improving their functions and resulting in a positive impact on mammalian longevity. Therefore, cardiovascular aging and longevity share common pathophysiological mechanisms, and delaying cardiovascular aging increases the likelihood of greater longevity (Pietri and Stefanadis, 2021). Many ewes are culled at a young age as the selection process requires that the generation interval be as short as possible. Therefore, heart diseases are not expected for young and fertile ewes, as in the current Katahdin population. However, Katahdin ewes are selected for increased fertility, and this selection may have some impact on the cardiovascular system as estrogen modulates multiple cardiovascular functions (Murphy, 2011). We did not find evidence of this possible positive or negative pleiotropic effect between reproductive performance and cardiovascular health in livestock. However, other traits such as residual feed intake seem to have some relationship with cardiac function (Munro et al., 2019). Moreover, gene expression in cardiac muscle from yaks and cattle suggests an involvement of cardiac function with high-altitude adaptation (Wang et al., 2021). Therefore, future studies should investigate whether ewe reproductive performance is associated with cardiovascular function, which would explain a possible effect on Katahdin ewe longevity.

### 4.7 Final considerations

We found genomic regions harboring several genes that may play key roles in growth, reproductive performance, health, milk production, and carcass quality in U.S. Katahdin sheep; these factors will directly impact breeders’ decision to retain ewes in the flock, thereby affecting ewe longevity. Ewe longevity differs from their life expectancy because ewes in commercial flocks are often culled prematurely due to many voluntary and involuntary reasons before their natural life expectancy. For instance, the current Katahdin ewe population had an average ALL of 1,102 days, i.e., on average, they were culled approximately 3 years old. Even healthy and fertile ewes rarely reach more than 7 years of productive life (McLaren et al., 2020; Hanna et al., 2023). This occurs because breeders annually replace some ewes with ewe-lambs to improve the genetic merit of their flocks. This practice contributes to reducing the generation interval on the female side and improves annual genetic gain. Therefore, the candidate genes identified for ewe productive longevity related to other important traits such as fertility and reproduction are unsurprising as these traits directly impact ewes’ ability to remain in a flock.

Longevity is still a poorly studied topic in sheep, with few studies reporting candidate genes directly associated with longevity traits. *FOXO3* (forkhead box O3) (Byun et al., 2011) and *IGF1R* (IGF1 receptor) (Byun et al., 2012) were two genes reported for sheep longevity, which were identified in candidate gene studies, while only one GWAS was reported for ewe longevity (Smitchger et al., 2024). Therefore, the current study provides more information by revealing novel genomic regions and candidate genes for longevity-related traits in sheep. Our genomic regions do not overlap with those previously reported for ewe longevity in other U.S. sheep breeds (Smitchger et al., 2024), which may be a consequence of the low-density SNP map used in both studies. Thus, we recommend that future studies use higher-density maps. Another hypothesis is the small polygenic effect and large environmental effects as longevity-related traits often show low heritability estimates (El-Saied et al., 2005; Borg et al., 2009; Zishiri et al., 2013; Lee et al., 2015). In a previous study with the current Katahdin population, we estimated heritability between 0.07 and 0.15 for these longevity indicators (Pinto et al., 2025b). It must be noted that the heritability estimates for longevity indicators may be moderate as culling reasons are considered in the analyses (Mekkawy et al., 2009). This could also improve GWAS analyses by identifying candidate genes most closely related to specific culling reasons. Therefore, NSIP and Katahdin breeders are encouraged to record the culling reasons to allow future genetic studies to separate voluntary or involuntary culling to improve the precision of association analyses.

## 5 Conclusion

We performed the first GWAS analyses for ewe longevity-related traits in U.S. Katahdin sheep and identified novel candidate genes and pathways associated with ewe longevity. Our results suggested that ewe longevity is under a complex polygenic control where candidate genes may be involved with prolificacy (*RORA*, *APOH*, *NLRP9*, *CKB*, and *HERC4*), a decline of the ovarian follicle pool (*GALNT13*, *TMEM150B*, and *BRSK1*), synthesis and release of some reproductive hormones (*SULT1B1*, *LEF1*, and *EIF5*), early pregnancy events (*ITGAV*, *HADH*, *ZNFX1*, *ZSCAN4*, *EPN1*, *FBXW8*, *NOS1, ST3GAL4*, *GFRA1*, and multiple lncRNAs), disease or syndromes affecting reproduction (*ADCY5* and *BBS9*), response to stress or pathological conditions (*ADCY5*, *HADH*, *ATRNL1*, *LEP*, *IL11*, *NLRP9*, *PRKCG*, *PRKCA*, *NEDD4L*, *FECH*, *CTNNA3*, *LRRTM3*, and zinc-finger proteins), growth performance (*GRID2*, *MED13L*, *DCPS*, and *LEP*), and carcass traits (*CMYA5* and *SETD3*). Moreover, our study found enriched pathways that may be influencing ewe longevity, such as oxytocin signaling and cardiac pathways. Taken together, this information suggests that Katahdin ewe longevity depends on a complex combination of voluntary and involuntary culling reasons, along with their underlying biological mechanisms. Identifying genes that are associated with early culling is an important step toward improving ewe longevity and enhancing the economic prosperity and sustainability of the U.S. sheep industry.

## Data Availability

The datasets presented in this article are not readily available because they are the property of the U.S. sheep producers, and the information in these datasets is commercially sensitive. Requests to access the datasets should be directed to the corresponding author.
