# Supplementary material for: Genome-wide association and functional annotation analyses reveal candidate genes and pathways associated with various ewe longevity indicators in U.S. Katahdin sheep
Source: Front Genet. 2025 Jul 3;16:1600587. doi: 10.3389/fgene.2025.1600587 (PMC12267044; doi:10.3389/fgene.2025.1600587)
Supplement: Supplementary file 2 [file DataSheet1.docx]

**SUPPLEMENTARY MATERIAL**

**Figure S1.** Frequency distribution of genotyped animals across years.


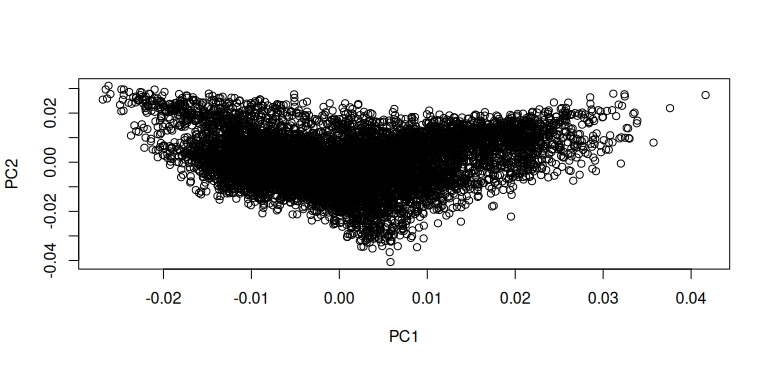


**Figure S2.** Graphical analysis of stratification by plotting the principal components 1 (PC1) and 2 (PC2).


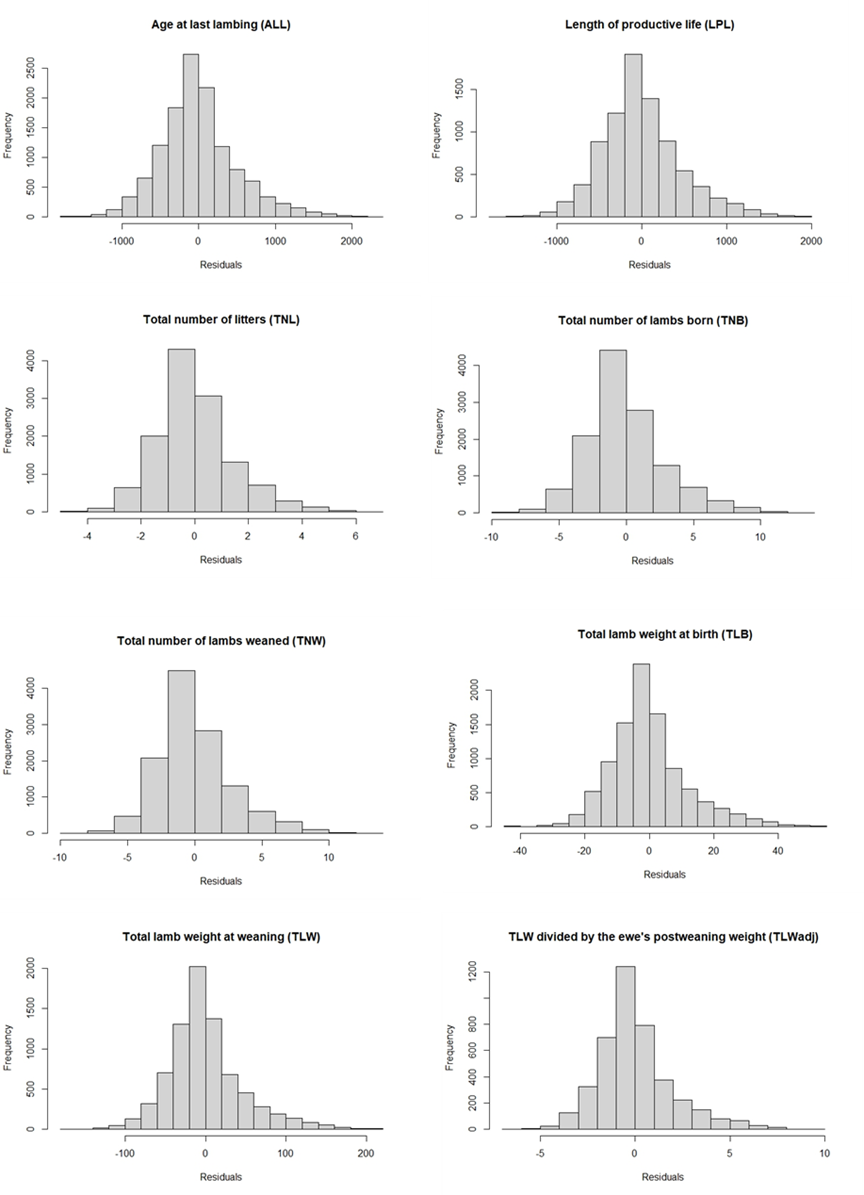


**Figure S3.**  Residual distribution of each analyzed trait.

**Table S1**. Significance threshold (p-value) for each *Ovis aries* chromosome (OAR) used in the GWAS analyses.

| OAR | p-value |
| --- | --- |
| 1 | 2.29 x 10^-04^ |
| 2 | 2.49 x 10^-04^ |
| 3 | 2.71 x 10^-04^ |
| 4 | 4.46 x 10^-04^ |
| 5 | 4.49 x 10^-04^ |
| 6 | 4.46 x 10^-04^ |
| 7 | 5.15 x 10^-04^ |
| 8 | 5.56 x 10^-04^ |
| 9 | 5.41 x 10^-04^ |
| 10 | 5.83 x 10^-04^ |
| 11 | 7.46 x 10^-04^ |
| 12 | 6.16 x 10^-04^ |
| 13 | 5.99 x 10^-04^ |
| 14 | 7.12 x 10^-04^ |
| 15 | 6.04 x 10^-04^ |
| 16 | 6.71 x 10^-04^ |
| 17 | 6.63 x 10^-04^ |
| 18 | 7.01 x 10^-04^ |
| 19 | 7.65 x 10^-04^ |
| 20 | 8.64 x 10^-04^ |
| 21 | 9.16 x 10^-04^ |
| 22 | 8.63 x 10^-04^ |
| 23 | 7.47 x 10^-04^ |
| 24 | 9.92 x 10^-04^ |
| 25 | 9.56 x 10^-04^ |
| 26 | 9.53 x 10^-04^ |
